# NMADTA: An R package for network meta-analysis of multiple diagnostic tests

**DOI:** 10.1017/rsm.2026.10097

**Published:** 2026-06-15

**Authors:** Xing Xing, Boyang Lu, Lifeng Lin, Qinshu Lian, James S. Hodges, Yong Chen, Haitao Chu

**Affiliations:** 1 Department of Biostatistics, Johns Hopkins Bloomberg School of Public Health, Baltimore, MD, USA; 2 Division of Biostatistics and Health Data Science, University of Minnesota, Minneapolis, MN, USA; 3 Department of Epidemiology and Biostatistics, The University of Arizona, Tucson, AZ, USA; 4 Department of Biostatistics, Genentech Inc., South San Francisco, CA, USA; 5 Department of Biostatistics, Epidemiology and Informatics, University of Pennsylvania, Philadelphia, PA, USA; 6 Statistical Research and Data Science Center, Pfizer Inc, New York, NY, USA

**Keywords:** bayesian methods, diagnostic tests, network meta-analysis, systematic review

## Abstract

Meta-analysis is a widely used statistical tool for estimating the diagnostic accuracy of tests across multiple studies. Existing methods and available R packages primarily focus on a single diagnostic test, typically under the assumption that all studies include a gold standard. Greater efficiency can be achieved by modeling multiple diagnostic tests together and drawing on studies with or without a gold standard reference test across diverse designs. To address this challenge, recent work has extended both the Bayesian hierarchical model and the Bayesian hierarchical summary receiver operating characteristic model to the framework of network meta-analysis of diagnostic tests, enabling simultaneous comparison of multiple tests when some data are missing. Despite the importance of these methods, their computational complexity has limited their broad application. This article introduces NMADTA, an R package that implements these models with user-friendly functions. The package allows researchers to evaluate the accuracy of multiple diagnostic tests simultaneously and provides comprehensive graphical displays of the results.

## Highlights

### What is already known?

- Meta-analysis methods for diagnostic test accuracy have evolved from simple summary receiver operating characteristic (SROC) models to hierarchical frameworks, such as hierarchical SROC and bivariate generalized linear mixed model.
- Existing approaches often require either all studies to include a gold standard or none, limiting their applicability to heterogeneous diagnostic settings.
- Current software tools mainly handle single-test meta-analyses and cannot simultaneously integrate multiple diagnostic tests with and without gold standards.

### What is new?

- We introduce the NMADTA R package for network meta-analysis (NMA) of multiple diagnostic tests, capable of handling both gold-standard and non-gold-standard studies.
- The package implements two flexible Bayesian models that accommodate study design heterogeneity and missing data.
- NMADTA provides user-friendly functions for data input, Markov chain Monte Carlo convergence checking, forest plot generation, and accuracy comparison across tests.

### Potential impact for RSM readers

- NMADTA addresses a critical methodological gap by enabling NMA of multiple diagnostic tests with or without a gold standard, accommodating real-world data heterogeneity.
- It provides a unified Bayesian framework that facilitates transparent evidence synthesis and improves decision-making in diagnostic test evaluation.
- The package empowers applied researchers and methodologists with accessible, reproducible, and extensible tools aligned with current reporting guidelines.

## 1 Introduction

Meta-analysis is a widely used statistical approach to synthesize results from multiple studies, which can improve statistical power and precision.^1^ ^,^ ^2^ Network meta-analysis (NMA) is an extension of traditional meta-analysis that allows the comparison of multiple treatments or diagnostic tests by combining direct evidence from individual studies with indirect evidence across studies.^3^ ^,^ ^4^ NMA has also been widely applied in practice, demonstrating its value across diverse clinical and methodological contexts.^5^ ^–^ ^7^ More recently, NMA has been extended to evaluate the accuracy of multiple diagnostic tests.^8^ ^,^ ^9^

Methodological developments in the meta-analysis of diagnostic tests have largely concentrated on evaluating and comparing the performance of one or multiple diagnostic tests across studies, either under the setting where a gold standard is consistently available or in the more challenging scenario where no study includes a definitive gold standard. Moses et al.^10^ introduced the summary receiver operating characteristic (SROC) curve to synthesize diagnostic test accuracy across studies. This approach was subsequently extended by Rutter and Gatsonis^11^ to incorporate between-study heterogeneity, leading to the hierarchical SROC (HSROC) model. Beyond the SROC and HSROC frameworks, Reitsma et al.^12^ proposed a bivariate linear mixed model and Chu and Cole^13^ developed a bivariate generalized linear mixed model (GLMM) that models true-positive and true-negative counts via binomial likelihoods, with sensitivity and specificity linked to correlated random effects through logit transformations. This bivariate GLMM was later generalized to accommodate arbitrary link functions^14^ and further extended to a trivariate GLMM to incorporate disease prevalence.^15^ More recently, Chen et al.^16^ introduced a hybrid multivariate random-effects model that integrates case-control and cohort studies while jointly modeling disease prevalence together with diagnostic sensitivity and specificity.

For meta-analysis of diagnostic tests without a gold standard, latent class models based on SROC or HSROC models were proposed by Walter et al.^17^ and Dendukuri et al.,^18^ respectively. Besides the SROC and HSROC approaches, Chu et al.^19^ used a random effect model to account for variation and correlation in prevalence, sensitivity, and specificity in the absence of a gold standard. Harbord et al.^20^ and Liu et al.^21^ unified GLMM and HSROC models for meta-analysis of diagnostic tests with and without a gold standard, respectively. However, both the HSROC and the GLMM approaches require that either all or none of the studies include a gold standard test. Furthermore, when comparing multiple diagnostic tests, existing approaches typically either perform separate meta-analyses for each test or apply meta-regression by incorporating test type as a covariate. Such strategies are often developed under relatively homogeneous study design assumptions, for example, requiring that all included studies adopt either a randomized comparative design or a non-comparative framework, which limits their applicability in more complex diagnostic networks.

A comprehensive overview of NMA-DTA methodology is provided by Veroniki et al.,^22^ who reviewed the available model classes and empirically assessed their operating characteristics. Within this broader landscape, the two Bayesian frameworks adopted in this article are, to the best of our knowledge, the only available approaches that can accommodate heterogeneous study designs and allow each study to contribute only a subset of candidate tests, while also permitting missing reference standards through explicit missing-data modeling. Specifically, Ma et al.^8^ introduced a Bayesian hierarchical network meta-analysis for diagnostic tests (MA-DT) model within a missing data framework. This approach is capable of synthesizing evidence from studies with diverse designs, including multiple test comparisons, randomized, and non-comparative studies. It accommodates both studies with and without a gold standard test and allows each study to contribute a distinct set of candidate tests. The model accounts for between-study heterogeneity and captures complex correlation structures among multiple diagnostic tests. Building on this framework, Lian et al.^9^ proposed a Bayesian HSROC model for NMA of diagnostic tests, which simultaneously compares multiple candidate tests while addressing missing data. Their model incorporates correlations among candidate tests and heterogeneity across studies, allows different studies to include different subsets of diagnostic tests, and offers flexibility in the choice of summary measures. Moreover, it provides estimates not only of overall disease prevalence and test-specific sensitivity and specificity, but also of additional accuracy metrics, such as positive and negative predictive values, consistent with reporting guidelines for diagnostic test accuracy studies.^23^ ^,^ ^24^

Many R packages support conventional meta-analysis,^25^ ^–^ ^28^ but only limited software is available for NMA, and even less for NMA of diagnostic tests. For diagnostic tests, the options are even more limited. The package HSROC ^29^ implements the HSROC model for meta-analysis of a single diagnostic test. Similarly, meta4diag ^30^ provides a graphical interface for the bivariate normal model for meta-analysis of a single diagnostic test and bamdit ^31^ offers models for NMA of a single diagnostic test. However, to date, there has been no publicly available and user-friendly software that provides a unified workflow for NMA of multiple diagnostic tests while simultaneously accommodating studies with and without a gold standard test.

This article introduces the package NMADTA, which extends existing methodological developments into a coherent, numerically stable, and user-friendly software framework. The package provides integrated functions for data preprocessing, model fitting, posterior inference, and result visualization for diagnostic test NMA using two established modeling approaches. The convergence of the Markov chain Monte Carlo (MCMC) routine can be assessed by examining the function’s outputs. The package provides functions to visualize comparisons of the accuracy of the different diagnostic tests in the meta-analysis. It also provides forest plots for 95% credible intervals of the study-specific performance of each test and relative effect sizes, visually displaying treatment effects and their comparisons.

This article is organized as follows. Section 2 presents an overview of the two Bayesian models implemented in the NMADTA package. Section 3 details the overall package design. Section 4 illustrates the package’s use with several examples and discusses its output data structure. Finally, Section 5 closes with suggested future improvements.

## 2 Bayesian hierarchical and HSROC models

This section introduces a Bayesian hierarchical model and a Bayesian HSROC model for NMA of diagnostic tests, which analyze a collection of *N* studies to compare *K* index tests simultaneously. Each study includes results of a subset of the complete collection of K+1 diagnostic tests ( $T_{0},T_{1},T_{2},\ldots ,T_{K}$ ), with $T_{0}$ being the gold standard test, which can be either present or absent in each study. Included studies can have different designs; all studies are viewed as if they used the crossover design and each subject is diagnosed by all *K* diagnostic tests and also the gold standard test. In each study, unavailable test outcomes are considered missing and assumed to be missing at random (MAR; i.e., given the observed data, failure to observe a datum does not depend on missing data). Both models make the conditional independence assumption, that is, candidate test outcomes are independent given true disease status and each model’s unknown parameters. The models presented in this section follow previously proposed methodological frameworks, and the underlying statistical formulations are identical. The primary contribution of this work is the software implementation rather than the development of new statistical models. In particular, we streamlined existing prototype code to enhance numerical stability and usability and integrated data pre-processing, model fitting, and posterior summarization into a unified and reproducible workflow. Although the hierarchical and HSROC formulations are closely related, they emphasize different parameterizations and are useful in slightly different practical settings. The hierarchical model is most convenient when the primary target is pooled sensitivity and specificity (and derived measures such as PPV/NPV) and when threshold effects are not expected to be the dominant driver of heterogeneity. In contrast, the HSROC formulation is often preferable when threshold differences across studies (e.g., different cutoffs or positivity criteria) are anticipated or when comparisons across operating points along the ROC curve are of interest. We provide a technical discussion of the relationship between the two formulations and related considerations in Section 4.2.

### 2.1 Likelihood specifications

Let $y_{ijk}$ be the binary outcome of test $T_{k}$ for subject *j* in study *i*, so $y_{ij0}$ is the outcome of the gold standard test. Let $\delta_{ijk}$ be a missing data indicator ( $\delta_{ijk}=0$ if missing, 1 if present). Let $\pi_{i}$ be the disease prevalence of study *i*, that is, $\pi_{i}=P(y_{ij0}=1),i=1,\ldots ,N$ . For k=1,…,K , let ${\text{Se}}_{ik}$ and ${\text{Sp}}_{ik}$ denote the sensitivity and specificity, respectively, for the $k^{\text{th}}$ test in study *i* ^9^:

$$\begin{array}{r} {\text{Se}}_{ik}=P(y_{ijk}=1|y_{ij0}=1)\text{ and }{\text{Sp}}_{ik}=P(y_{ijk}=0|y_{ij0}=0). \end{array}$$

If a gold standard test is present, the outcome for test *k* applied to subject *j* in study *i* is $Y_{ijk}∼\text{Bernoulli}({\text{Se}}_{ik})$ , k>0 if the subject is truly diseased, that is, $y_{ij0}=1$ , and $Y_{ijk}∼\text{Bernoulli}(1-{\text{Sp}}_{ik})$ , k>0 , if $y_{ij0}=0$ . Let $y_{ij}^{c}=(y_{ij}^{o},y_{ij}^{m})$ denote the vector containing outcomes of the complete test set for subject *j* in study *i*, where superscripts *o* and *m* denote observed and missing outcomes. Let $K_{i}$ be the set of index tests included in study *i*.

If the gold standard test is observed in study *i*, the likelihood contribution of subject *j*’s outcomes is^9^

(2.1) $$\begin{array}{r} \begin{array}{rl} l_{ij1} & =\pi_{i}\prod_{k\in K_{i}}({\text{Se}}_{ik}{)}^{y_{ijk}^{o}}(1-{\text{Se}}_{ik}{)}^{\left(1-y_{ijk}^{o}\right)}\\ & \times (1-\pi_{i})\prod_{k\in K_{i}}(1-{\text{Sp}}_{ik}{)}^{y_{ijk}^{o}}({\text{Sp}}_{ik}{)}^{\left(1-y_{ijk}^{o}\right)}. \end{array} \end{array}$$

When the gold standard test is observed, the disease status of subject *j* in study *i* is known through $y_{ij0}$ . Conditional on this status, the likelihood contribution of the observed index test outcomes can be written as the product of the likelihood over the two possible disease states. Specifically, diseased subjects occur with probability $\pi_{i}$ , for whom the joint binomial likelihood is the product of a function of sensitivities; while non-diseased subjects occur with probability $1-\pi_{i}$ , for whom the joint likelihood is the product of a function of specificities.

If the gold standard test is missing in study *i*, the likelihood contribution of subject *j*’s observed outcomes is^9^

(2.2) $$\begin{array}{r} \begin{array}{rl} l_{ij0} & =\pi_{i}\prod_{k\in K_{i}}({\text{Se}}_{ik}{)}^{y_{ijk}^{o}}(1-{\text{Se}}_{ik}{)}^{\left(1-y_{ijk}^{o}\right)}\\ & +(1-\pi_{i})\prod_{k\in K_{i}}(1-{\text{Sp}}_{ik}{)}^{y_{ijk}^{o}}({\text{Sp}}_{ik}{)}^{\left(1-y_{ijk}^{o}\right)}. \end{array} \end{array}$$

If the gold standard test is not observed, the disease status of subject *j* in study *i* is not observed. Then, the likelihood contribution of the observed index test results follows a two-component mixture, weighted by the study-specific prevalence.

### 2.2 A Bayesian hierarchical model for NMA of diagnostic tests

#### 2.2.1 Hierarchical model

To allow and capture heterogeneity between studies in prevalence, sensitivities, specificities, and correlations among them, we include multivariate random effects in the meta-regression model and write^8^

(2.3) $$\begin{array}{r} \pi_{i}=\Phi (\eta +\epsilon_{i}),{\text{Se}}_{ik}=\Phi (\alpha_{k}+\mu_{ik}),{\text{Sp}}_{ik}=\Phi (\beta_{k}+v_{ik}),i=1,\ldots ,N,k=1,\ldots ,K, \end{array}$$

where Φ(⋅) is the standard normal cumulative distribution function. The parameter η is a fixed effect for prevalence and the parameters $\alpha_{k}$ and $\beta_{k}$ are fixed effects for the sensitivity and specificity of $T_{k}$ , respectively. The $\epsilon_{i}$ is the study-specific random effect for prevalence in study *i*, and $\mu_{ik}$ and $\nu_{ik}$ are the study-specific random effects for the sensitivity and specificity of test $T_{k}$ in study *i*, respectively, capturing between-study heterogeneity and inducing correlation among prevalence, sensitivity, and specificity across tests.

We assume within-study random effects follow the multivariate normal distribution to account for dependency between test parameters and correlation between prevalence and test parameters if applicable, that is,

$$\begin{array}{r} \theta_{i}=(\epsilon_{i},\mu_{i1},\nu_{i1},\ldots ,\mu_{iK},\nu_{iK}{)}^{⊤}∼MVN(0,\Sigma ),i=1,\ldots ,N \end{array}$$

as in Ma et al.^8^ The covariance matrix Σ=SΩS , where S is a diagonal matrix; its diagonal entries are standard deviations describing between-study heterogeneity, while Ω has diagonal entries equal to 1 and off-diagonal entries measuring correlations between prevalence and sensitivities and specificities.

#### 2.2.2 Prior specifications

The precision matrix $\Sigma^{-1}$ follows a Wishart( R,v ) distribution, where the degrees of freedom parameter v=2K+1 , the dimension of Σ . The matrix R acts as a scale (inverse prior mean) for $\Sigma^{-1}$ , so it determines the prior magnitude of between-study variability and the strength of prior dependence across random effects. Since the Wishart distribution has support only on symmetric positive-definite matrices, placing a Wishart prior on the precision matrix $\Sigma^{-1}$ guarantees that the implied covariance matrix Σ is also positive definite. Consequently, all required covariance constraints, such as $a_{ij}^{2}\leq a_{ii}a_{jj}$ for all i≠j , are automatically satisfied. The choice of R can vary according to different situations; different choices of R give priors containing different levels of information. Section 4.2 describes different choices of priors on R available in NMADTA. Vague normal priors with mean 0 and variance 10 are placed on $\eta ,\alpha_{k}$ , and $\beta_{k}$ ( k=1,…,K ).

#### 2.2.3 Posterior distribution

Given the likelihood and prior specifications above, the posterior can be written as^8^

$$\begin{array}{r} \begin{array}{rl} & P\;\left(\eta ,\alpha ,\beta ,\{\epsilon_{i},\mu_{i},\nu_{i}{\}}_{i=1}^{N}\;|\;y\right)\propto \\ & \prod_{i=1}^{N}\left[\prod_{j=1}^{J_{i}}(l_{ij1}{)}^{\delta_{i0}}(l_{ij0}{)}^{1-\delta_{i0}}|\Sigma {|}^{-1/2}e^{-\frac{1}{2}\theta_{i}^{\prime }\Sigma^{-1}\theta_{i}}\right]\times \left\{\prod_{k=1}^{K}f(\alpha_{k})f(\beta_{k})\right\}f(\eta )f(\Sigma ), \end{array} \end{array}$$

where $J_{i}$ is the number of subjects in study *i*, and $l_{ij1}$ and $l_{ij0}$ are defined above in equations (2.1) and (2.2).

Posterior medians for prevalence, sensitivity, and specificity of $T_{k}$ can be estimated using the MCMC sampling and these definitions: π=Φ(η) , ${\text{Se}}_{k}=\Phi (\alpha_{k})$ , and ${\text{Sp}}_{k}=\Phi (\beta_{k})$ . Posterior medians of other measures, such as positive and negative predictive values (PPV and NPV) and positive and negative likelihood (LR + and LR − ) for each candidate test, can also be calculated using

$$\begin{array}{r} \begin{array}{rl} {\text{PPV}}_{k}=\frac{{\text{Se}}_{k}\pi }{{\text{Se}}_{k}\pi +(1-{\text{Sp}}_{k})(1-\pi )}; & \;{\text{NPV}}_{k}=\frac{{\text{Sp}}_{k}(1-\pi )}{{\text{Sp}}_{k}(1-\pi )+(1-{\text{Se}}_{k})\pi };\\ {\text{LR}}_{+k}=\frac{{\text{Se}}_{k}}{1-{\text{Sp}}_{k}}; & \;{\text{LR}}_{-k}=\frac{1-{\text{Se}}_{k}}{{\text{Sp}}_{k}}. \end{array} \end{array}$$

The SROC curve is obtained by using equation (2.3) to obtain a regression line of $\alpha_{k}$ on $\beta_{k}$ ^32^ to estimate the median probit transformed sensitivity given several values of 1− specificity:

$$\begin{array}{r} {\text{Se}}_{k}=\Phi \left\{-\alpha_{k}+\frac{\Sigma_{3k-1,3k}}{\Sigma_{3k,3k}}(\Phi^{-1}\left(1-{\text{Sp}}_{k})-\beta_{k}\right)\right\}, \end{array}$$

where Σ is specified above as the covariance matrix, $\Sigma_{3k-1,3k}$ is the correlation between ${\text{Se}}_{k}$ and ${\text{Sp}}_{k}$ , and $\Sigma_{3k,3k}$ captures the variance of ${\text{Sp}}_{k}$ .

### 2.3 A Bayesian HSROC models for NMA of diagnostic tests

#### 2.3.1 The HSROC model

The HSROC model has three layers that capture variability within a study, variation between studies, and correlations among tests in the same study.

We assume that the outcomes of all tests are dichotomous. Within a study, let the latent variable $Z_{ijk}$ follow a normal distribution^9^:

$$\begin{array}{r} \begin{array}{c} Z_{ijk}∼\left\{ \begin{array}{lcl} N\left(-\alpha_{ik}/2,\exp(-\beta_{k}/2)\right) & \text{for} & y_{ij0}=0,\\ N\left(\alpha_{ik}/2,\exp(\beta_{k}/2)\right) & \text{for} & y_{ij0}=1. \end{array}\right. \end{array} \end{array}$$

If $Z_{ijk}$ is greater than the study-specific cutoff value $\theta_{ik}$ , subject *j* in study *i* has a positive result for $T_{k}$ ; otherwise, the result is negative.

The parameter $\alpha_{ik}$ measures the difference between the distributions of $Z_{ijk}$ when disease is present versus absent in study *i*. The shape parameter $\beta_{k}$ measures the difference in the variance in the outcomes of diseased and non-diseased populations. The $\beta_{k}$ ’s are assumed to be the same across all studies because otherwise the model is not identified in typical datasets. Although we use the same notation α and β in both model formulations to denote test-specific fixed effects, they enter the models through different parameterizations. In the SROC-based model, $\alpha_{k}$ and $\beta_{k}$ act directly on the link functions of sensitivity and specificity, whereas in the HSROC formulation, the fixed effects are reparameterized into accuracy and threshold components and enter through different linear predictors. Thus, while the underlying notion of test-specific fixed effects is shared, their interpretations depend on the chosen parameterization.

With these assumptions, test $T_{k}$ in study *i* has sensitivity and specificity^9^:

$$\begin{array}{r} {\text{Se}}_{ik}=\Phi \left(-\frac{\theta_{ik}-\alpha_{ik}/2}{\exp(\beta_{k}/2)}\right),{\text{Sp}}_{ik}=\Phi \left(\frac{\theta_{ik}+\alpha_{ik}/2}{\exp(-\beta_{k}/2)}\right). \end{array}$$

Let $\theta_{i0}$ be the cutoff for the gold standard in study *i* and let the true disease status $D_{ij}$ be positive if the latent variable $Z_{ij0}>\theta_{i0}$ and negative otherwise. This accounts for heterogeneity of prevalence between studies; the study-specific prevalence can be written as $\pi_{i}=\Phi (-\theta_{i0})$ .

For each study, let $\alpha_{i}=(\alpha_{i1},\ldots ,\alpha_{iK}{)}^{⊤}$ and $\theta_{i}=(\theta_{i0},\theta_{i1},\ldots ,\theta_{iK}{)}^{⊤}$ ( i=1,2,…,N ) be mutually independent.^11^ ^,^ ^18^ Let the study-specific cutoff values $\theta_{i}$ and values $\alpha_{i}$ follow multivariate normal distributions to account for heterogeneity between studies and correlations between different index tests^9^:

$$\begin{array}{r} \begin{array}{rl} \left(\begin{array}{c} \theta_{i0}\\ \theta_{i1}\\ \vdots \\ \theta_{iK} \end{array}\right) & \overset{\text{iid}}{∼}\text{MVN}\left(\begin{array}{c} \Theta =\left(\begin{array}{c} \Theta_{0}\\ \Theta_{1}\\ \vdots \\ \Theta_{K} \end{array}\right),\Sigma_{\Theta }=\left(\begin{array}{cccc} \Sigma_{0}^{2} & \Sigma_{01} & \ldots & \Sigma_{0K}\\ \Sigma_{01} & \Sigma_{1}^{2} & \ldots & \vdots \\ \vdots & \vdots & \ddots & \vdots \\ \Sigma_{0K} & \ldots & \ldots & \Sigma_{K}^{2} \end{array}\right) \end{array}\right)\;\text{and}\\ \left(\begin{array}{c} \alpha_{i1}\\ \alpha_{i2}\\ \vdots \\ \alpha_{iK} \end{array}\right) & \overset{\text{iid}}{∼}\text{MVN}\left(\begin{array}{c} \Lambda =\left(\begin{array}{c} \Lambda_{1}\\ \Lambda_{2}\\ \vdots \\ \Lambda_{K} \end{array}\right),\Sigma_{\Lambda }=\left(\begin{array}{cccc} \tau_{1}^{2} & \tau_{12} & \ldots & \tau_{1K}\\ \tau_{12} & \tau_{2}^{2} & \ldots & \vdots \\ \vdots & \vdots & \ddots & \vdots \\ \tau_{1K} & \ldots & \ldots & \tau_{K}^{2} \end{array}\right) \end{array}\right),\;i=1,\ldots ,N, \end{array} \end{array}$$

where the covariance matrices $\Sigma_{\Theta }$ and $\Sigma_{\Lambda }$ are positive definite with diagonal entries capturing between-study variability in the cutoff and accuracy values, and off-diagonal entries measuring covariance between tests of pairs of cutoff and accuracy values, respectively. Also, $\Sigma_{\Theta }$ and $\Sigma_{\Lambda }$ are independent and jointly determine the correlation between index tests’ sensitivities and specificities.^9^ This model assumes that $\Sigma_{\Theta }$ and $\Sigma_{\Lambda }$ are unstructured, and the data will permit inferences about these correlations.

#### 2.3.2 Prior specifications

We assume that the mean cutoff values $\Theta_{k}$ and accuracy values $\Lambda_{k}$ , k=1,2,…,K have vague normal priors with large variance. The shape parameters $\beta_{k}$ have a Uniform $\left(b_{1},b_{2}\right)$ prior where $b_{1}$ and $b_{2}$ depend on prior knowledge about the index test *k* and $\left(b_{1},b_{2}\right)$ should allow all possible $\beta_{k}$ .

We decompose the covariance matrix $\Sigma_{\Theta }$ of the cutoff values as diag( S ) ⋅R⋅ diag( S ), where diag( S ) is a diagonal matrix with diagonal entries $S=(s_{0},s_{1},\ldots ,s_{K}$ ) and R is a (K+1)×(K+1) matrix that captures the correlations between cutoff values. This decomposition, using a separation technique, can simplify specification and simplify calculations by not forcing R defined above to be a correlation matrix,^33^ as follows. The cutoff values have standard deviations $\Sigma_{k}=\sqrt{s_{k}^{2}\cdot R_{k,k}}$ ( k=0,1,…,K ), while the correlation matrix can be written as ${\text{Corr}}_{\Theta }=\text{diag}(R{)}^{-1/2}\cdot R\;\text{diag}(R{)}^{-1/2}$ , so that these items are identified even though S and R are not. Similarly, decompose the covariance matrix of the accuracy values $\Sigma_{\Lambda }$ as $\Sigma_{\Lambda }=\text{diag}(P)\cdot \Omega \cdot \text{diag}(P)$ , where $P=(p_{1},\ldots ,p_{K})$ is a diagonal matrix and the standard deviations and correlation matrix for the accuracy values are $\tau_{k}=\sqrt{p_{k}^{2}\cdot \Omega_{k,k}}$ and ${\text{Corr}}_{\Lambda }=\text{diag}(\Omega {)}^{-1/2}\cdot \Omega \cdot \text{diag}(\Omega {)}^{-1/2}$ , respectively. Let each element of log⁡(P) and log⁡(S) follow an $N(\eta ,\zeta^{2})$ prior, where $\zeta^{2}$ is a variance, and assign values to η and $\xi^{2}$ based on prior knowledge about their standard deviations. When prior information does not suggest different magnitudes of between-study variability for cutoff and accuracy components, we can use the same weakly informative hyperparameters. Let R and Ω follow Inverse-Wishart priors $\text{IW}(I_{K+1},K+2)$ and $IW(I_{K},K+1),$ respectively, where $I_{K+1}$ and $I_{K}$ are identity matrices of dimensions K+1 and K, respectively. These prior specifications imply that the marginal priors of all correlation parameters are approximately uniform. Studies including all tests contribute to the full matrices $\Sigma_{\Theta }$ and $\Sigma_{\Lambda }$ while studies with missing tests contribute only to submatrices of them. We note that identifiability can be challenging in Bayesian NMA models for diagnostic tests, particularly when the network is sparse (e.g., many studies include only a subset of tests) or when the reference gold standard is frequently missing. In such settings, posterior inferences for variance–covariance components and correlation structures may be more sensitive to prior choices, and MCMC mixing can be slower. We therefore recommend routine convergence diagnostics and prior sensitivity analyses in case studies.

#### 2.3.3 Posterior distribution

Using the priors and likelihood functions specified above, the joint posterior distribution can be written as^9^

$$\begin{array}{r} \begin{array}{rl} & L(\theta_{i},\alpha_{i},\beta ,\Theta ,\Sigma_{\Theta },\Lambda ,\Sigma_{\Lambda }|y^{o})\\ & \propto \prod_{i}\left[\prod_{j}((l_{ij1}{)}^{\delta_{i0}}(l_{ij0}{)}^{1-\delta_{i0}})\times |\Sigma_{\Theta }{|}^{-1/2}e^{-\frac{1}{2}(\theta_{i}-\Theta {)}^{{}^{\prime }}\Sigma_{\Theta }^{-1}(\theta_{i}-\Theta )}|\Sigma_{\Lambda }{|}^{-1/2}e^{-\frac{1}{2}(\alpha_{i}-\Lambda {)}^{{}^{\prime }}{\Sigma_{\Lambda }}^{-1}(\alpha_{i}-\Lambda )}\right]\\ & \times f_{\Theta }(\Theta )f_{\Lambda }(\Lambda )f_{P}(P)f_{S}(S)f_{R}(R)f_{\Omega }(\Omega )f_{\beta }(\beta ). \end{array} \end{array}$$

The total number of degrees of freedom of this model varies with both the number of index tests included in a study and whether a gold standard test is included. When results from a gold standard test are available, a study contributes $2^{\left(K_{i}+1\right)}-1$ degrees of freedom; otherwise, it contributes $2^{K_{i}}-1$ degrees of freedom, where $K_{i}$ is the number of candidate tests included in study *i*. Thus, the total degrees of freedom could vary from 3N to $N\times (2^{K+1}-1)$ depending on the designs of the *N* studies. Since the total number of parameters is at least $K^{2}+5K+2$ , the minimum number of studies *N* required to estimate this model without setting informative priors is between $\frac{K^{2}+5K+2}{2^{K+1}-1}$ and $\frac{K^{2}+5K+2}{3}$ depending on the study designs.^9^

We can sample from the joint posterior distribution using MCMC sampling algorithm implemented in JAGS.^34^ The overall prevalence of the disease ( Π ) and the sensitivity and specificity of each test can be written as^9^

$$\begin{array}{r} \begin{array}{c} \Pi =\Phi \left(-\Theta_{0}\right),{\text{Se}}_{k}=\Phi \left(-\frac{\Theta_{k}-\Lambda_{k}/2}{\exp(\beta_{k}/2)}\right),{\text{Sp}}_{k}=\Phi \left(\frac{\Theta_{k}+\Lambda_{k}/2}{\exp(-\beta_{k}/2)}\right). \end{array} \end{array}$$

We will use medians and equal-tailed credible intervals of these posterior samples for inferences.

Other accuracy measures, such as a diagnostic test’s PPV and NPV, can also be calculated from the MCMC samples.^9^ The SROC curve can be derived using posterior samples of Λ , Θ, and β . As in Rutter and Gatsonis,^11^ we can use equation (2.4) to express the test-specific sensitivities in terms of given values of specificities by writing $\Theta_{k}$ as an expression of $\Lambda_{k}$ , $\beta_{k},$ and $Sp_{k}$ and eliminating $\Theta_{k}$ in the expression of ${\text{Se}}_{k}$ ^9^:

$$\begin{array}{r} {\text{Se}}_{k}=\Phi \left\{-\frac{\Phi^{-1}({\text{Sp}}_{k})\exp(-\beta_{k}/2)-\Lambda_{k}}{\exp(\beta_{k}/2)}\right\}. \end{array}$$

## 3 Package design and implementation

As R has become one of the most widely used platforms for statistical computing and visualization, we developed the NMADTA package entirely within the R ecosystem to ensure accessibility and reproducibility for applied researchers in evidence synthesis. The package implements Bayesian hierarchical and HSROC models for NMA of multiple diagnostic tests described in Section 2.

To handle computationally intensive Bayesian estimation, the core MCMC sampling procedures are executed via Just Another Gibbs Sampler (JAGS) through the rjags interface. JAGS was chosen because it is (i) widely adopted in the R community, (ii) offers transparent model specification in a BUGS-like syntax, (iii) ensures compatibility across operating systems, and (iv) provides stable convergence diagnostics via the coda package. This design allows users to directly inspect and modify the underlying model files to customize prior settings or model structures as needed.

Within NMADTA, the primary functions nmadt.hierarchical and nmadt.hsroc implement NMA for diagnostic test accuracy, while nmadt.hierarchical.MNAR and nmadt.hsroc.MNAR provide sensitivity analysis under the MNAR assumptions. In particular, nmadt.hierarchical and nmadt.hierarchical.MNAR implement the hierarchical method (model 1) and nmadt.hsroc and nmadt.hsroc.MNAR implement the HSROC method (model 2). These functions return S3 objects of class nmadt, allowing for consistent downstream processing. The commands in each of the following sections may take 10–30 minutes on an Intel 2.60 GHz processor. The actual run time depends on the complexity of the network, the setup of the meta-analysis, and the user’s processor. To clarify the missing-not-at-random (MNAR) assumption considered here, we emphasize that MNAR refers to outcome-dependent selection mechanisms operating at the test or study level within the diagnostic evidence network, in which the probability that a diagnostic test or study is included or reported depends on its underlying (possibly unobserved) accuracy parameters, such as sensitivity and specificity. In the context of diagnostic test NMA, this may arise when tests or studies with more favorable performance are more likely to be published or fully reported, while tests or studies with poorer performance are less likely to appear in the evidence network.

Two practical mechanisms are particularly relevant. First, selective study conducts or publication: studies evaluating tests with low sensitivity or specificity may be less likely to be conducted or published. Second, selective reporting within multi-test studies: results for poorly performing tests may be omitted, leading to incomplete availability of accuracy data. These mechanisms induce dependence between missingness and the unobserved true accuracy parameters and therefore fall within the MNAR framework.

Corresponding S3 methods, including print.nmadt, summary.nmadt, and plot.nmadt, provide standardized summaries and visualizations. The summary.nmadt method produces a structured table of posterior means, standard deviations, medians, and 95% equal-tail credible intervals for sensitivity, specificity, prevalence, predictive values, and likelihood ratios, ensuring a uniform reporting format. The print.nmadt method offers a concise textual summary suitable for console output, while plot.nmadt automatically generates graphical diagnostics, such as SROC curves, contour plots, posterior density plots, and hierarchical forest plots depending on the model type. This modular and standardized design facilitates reproducibility, readability, and interoperability across analyses conducted using the NMADTA package. To ensure transparency and flexibility, the entire plotting subsystem is implemented in pure R without external dependencies. Moreover, all Bayesian model definitions are stored as plain-text scripts within an internal JAGS models, enabling fully reproducible and platform-independent access to JAGS-based hierarchical and HSROC models.

Although the current implementation is optimized for use within R, future extensions could incorporate alternative backends, such as Stan or TensorFlow Probability to further improve computational efficiency and scalability. This limitation and the rationale for using JAGS as the current sampling engine are explicitly acknowledged in the manuscript.

Users can download the source file of NMADTA at https://CRAN.R-project.org/package=NMADTA. The NMADTA package depends on the R packages rjags,^34^ coda,^35^ and ggplot2.^36^ Users need to install JAGS separately to run the MCMC algorithms. This section introduces basic usage of the NMADTA package.

## 4 Using the R package NMADTA

NMADTA provides a unified statistical framework for simultaneously comparing multiple diagnostic tests across studies, even when head-to-head comparisons are incomplete. This approach allows estimation of the relative and absolute accuracy of each test, synthesizing evidence more efficiently than pairwise meta-analysis. To demonstrate its functionality and visualization capabilities, we present illustrative examples in the following sections, showcasing how NMADTA performs hierarchical and HSROC modeling, summarizes posterior results, and generates plots. For reproducibility, all analyses and code examples presented in this section were conducted on a Windows 10 x64 platform (build 22631, x86-64 architecture) using R version 4.3.2, JAGS version 4.3.2, and NMADTA version 0.1.3. It should be noted that, due to differences in JAGS random number generation during posterior MCMC sampling, minor numerical discrepancies may occur across different Mac environments. In contrast, stable reproducibility of the results was verified across multiple Windows devices.

### 4.1 Data structure for network meta-analysis

To illustrate, we first provide a brief introduction to the necessary data structures for the package NMADTA, with the dataset dat.kang as an example. The dataset dat.kang contains 12 studies comparing the accuracy of diagnostic tests for deep vein thrombosis (DVT).^37^ Among these 12 studies, 4 compared the D-dimer test to venography (the gold standard test), 3 compared ultrasonography to venography, and 5 compared the D-dimer test to ultrasonography.^37^ None of the studies simultaneously compared all three tests. However, the NMADTA software fully accommodates multi-test diagnostic studies through the joint likelihood modeling within studies, rather than restricting the analysis to pairwise comparisons. The dataset’s first few rows are shown below. The column sid contains numerical IDs for the 12 studies, and deltai indicates whether diagnostic test *i* is included in each study (delta0 denotes the presence of the gold standard test). For example, Study 1 compares $T_{1}$ with the gold standard test $T_{0}$ . The column Ti denotes the test outcome for test *i* (Ti=999 when test *i* is not included in the study) and the column *n* denotes the number of subjects that have the corresponding outcome in each row’s study. In the current implementation, NMADTA parses the input dataset by column position rather than by column names; therefore, the input data must follow a fixed column order. Specifically, the dataset should contain exactly 2K+4 columns arranged as follows: the first column is the study identifier (sid); columns 2 to K+2 are missingness indicators ( $\delta_{i}$ ) indicating whether diagnostic test *i* is included in each study ( $\delta_{0}$ ); columns K+3 to 2K+3 record the observed test outcomes ( $T_{i}$ ); and the final column contains the corresponding counts (*n*), representing the number of subjects sharing the same outcome pattern within each study. Although column names are not enforced by the software, variables must appear in the specified column positions and follow the required coding conventions. Each row represents a joint outcome pattern across tests observed in a study, and *n* is the number of subjects with that pattern. A study with *m* binary tests contributes up to $2^{m}$ rows; for example, Study 1 has $2^{2}=4$ rows for $\left(T_{0},T_{1}\right)$ . When $\delta_{0}=1$ , these patterns map to the index test’s 2×2 table against $T_{0}$ (TP/FN/FP/TN). When $\delta_{0}=0$ , only joint patterns among observed index tests are needed, and the model handles latent disease status and within-study dependence; no additional 2×2 test-by-test contingency tables are required.

**Figure:**
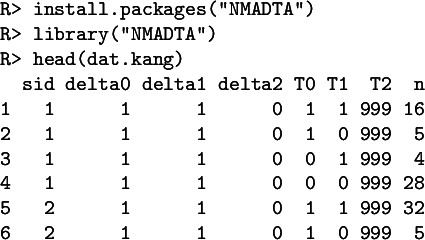

### 4.2 Bayesian network meta-analysis

Although the SROC- and HSROC-based formulations differ in parameterization, they are theoretically equivalent up to reparameterization under standard frequentist settings without missing reference standards. However, with Bayesian implementation, priors are specified in different scales and establishing theoretical equivalence is not straightforward and beyond the scope of this software article. Both frameworks assume an underlying multivariate latent structure for sensitivities and specificities and induce a summary ROC curve, but they differ in how test performance indexes are modeled: the SROC formulation directly models sensitivity and specificity, whereas the HSROC formulation decomposes performance into accuracy and threshold components. In practice, the SROC-based model facilitates direct estimation and interpretation of pooled sensitivity and specificity, while the HSROC parameterization can be advantageous when between-study heterogeneity is largely driven by implicit threshold differences across studies or when comparisons are of interest across different operating points of the ROC curve. Neither formulation is specifically designed to address sparse contingency tables (except implemented using Bayesian approaches), and any differences in numerical stability are primarily related to parameterization and estimation algorithms rather than fundamental theoretical distinctions. In the presence of missing reference standards, both models are affected by the extent of missingness, and appropriate modeling of the missing-data mechanism is critical; in NMADTA, this is handled through explicit MNAR modeling, rather than relying on differences between SROC and HSROC formulations to mitigate missingness-related bias.

#### 4.2.1 Function nmadt.hierarchical() for the regular Bayesian hierarchical model

The arguments of the function nmadt.hierarchical are as follows:

**Figure:**
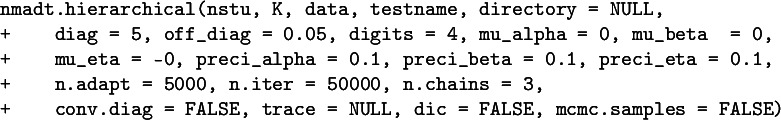
Long description.

Users need to input a dataset with structure similar to dat.kang, described above. The arguments nstu and K specify the number of studies and the number of candidate diagnostic tests included in the dataset. The argument data is the dataset to be meta-analyzed. The argument testname is a string vector that specifies the names of the candidate diagnostic tests. The argument directory is a string that specifies the directory to save trace plots or coda samples when trace or mcmc.samples are set to TRUE. The arguments diag and off_diag specify the values of diagonal and off-diagonal entries of the scale matrix R of the precision matrix Σ . The arguments diag and off_diag determine the scale matrix R in the Wishart prior for the precision matrix, $\Sigma^{-1}∼\text{Wishart}(R,\nu )$ . In NMADTA, R is constructed with diagonal entries equal to diag and off-diagonal entries equal to off_diag. Larger values of diag typically imply a strong prior shrinkage, favoring smaller between-study variability in prevalence, sensitivity, and specificity, whereas off_diag controls the strength of prior dependence among these random effects (e.g., correlations between sensitivity and specificity and their association with prevalence). The default setting (diag=5, off_diag=0.05) is weakly informative and works well in most applications; if strong correlations are expected, users may increase off_diag, and if substantially larger heterogeneity is anticipated, users may decrease diag. We recommend assessing sensitivity of posterior summaries to these choices in conjunction with standard MCMC convergence diagnostics. The argument digits is used to specify the number of digits to the right of the decimal point to keep for the results; the default is digits = 4.

We assume that the priors for α , β , and η follow a vague normal distribution with mean 0 and variance 10 (the default), which corresponds to 95% prior credible intervals of approximately (0,1) for test specific prevalence, sensitivity, and specificity.^8^

The arguments n.adapt, n.iter, n.burnin, n.chains, and n.thin specify the MCMC algorithm run by rjags. The argument n.adapt is the number of iterations for adaptation (the default is 5,000). The argument n.iter is the number of iterations in each MCMC chain, and n.burnin is the number of burn-in iterations at the beginning of each chain that are not saved. The argument n.chains=3 (the default) is the number of MCMC chains. The argument n.thin is the thinning rate for each chain, which can be used to save memory and computation time when n.iter is large. For example, the algorithm saves only one sample in every $n^{\text{th}}$ iteration, where *n* is given by n.thin.

The argument conv.diag specifies whether to compute potential scale reduction factors (PSRFs) proposed by Gelman and Rubin^38^ for convergence diagnostics. The argument trace is a string vector describing the subset of quantities for which trace plots can be drawn, which can include prevalence ("prev"), sensitivity ("Se"), specificity ("Sp"), positive and negative predictive values ("ppv" and "npv", respectively), positive likelihood ("LRpos"), and negative likelihood ("LRneg"). Trace plots for the specified quantities are saved in the user-specified directory as .png files. Both PSRF statistics and trace plots can be used to judge whether the MCMC chains converged eventually. Finally, if dic = TRUE, the function will output the deviance information criterion (DIC) statistic proposed by Spiegelhalter et al.^39^ The argument mcmc.samples is a logical value indicating whether the coda samples generated in the meta-analysis are saved in the output object. By specifying the argument mcmc.samples = TRUE in nmadt.hierarchical, nmadt.hierarchical.MNAR, nmadt.hsroc, and nmadt.hsroc.MNAR, the MCMC samples are saved in the output objects. Users can then use packages such as mcmcse ^40^ to determine whether the chains were run long enough to give sufficiently accurate estimates of posterior quantities.

The function nmadt.hierarchical returns a list with the raw output for graphing the results, the effect size estimates, including the posterior mean, standard deviation, median, and a 95 % equal-tailed credible interval for the median.

**Figure:**
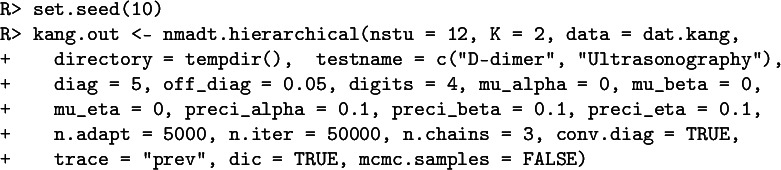
Long description.

The following messages are output as the function runs:

**Figure:**
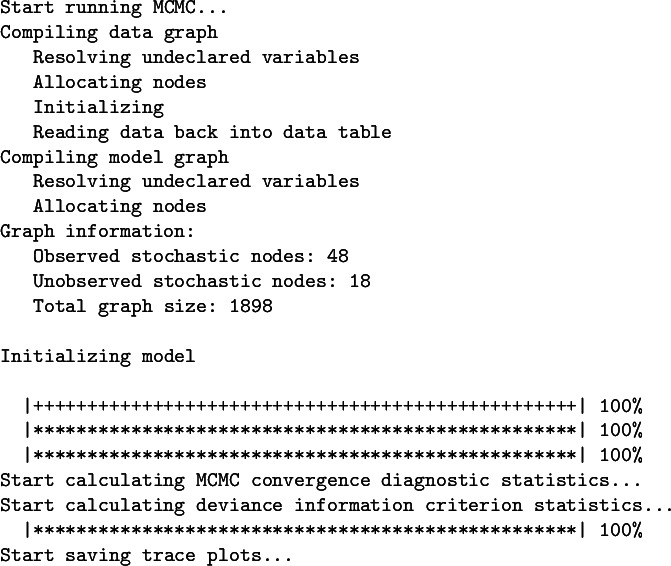
Long description.

When the proposed model is compiled, it requires an initial sampling phase during which the samplers adapt their behavior to maximize their efficiency. If the warning “adaptation incomplete” occurs, the users might need to increase n.adapt to make the process reach its maximum efficiency.

The results are saved in the list object kang.out. It contains the raw output for prevalence, sensitivity, specificity, positive and negative predictive values, positive likelihood, negative likelihood, and DIC. The trace plots are saved in the user-specified directory. The effect size names can be used to display the estimates. For example, the estimates of sensitivities and specificities (posterior mean and standard deviation, and posterior median and 95% credible intervals) can be displayed as

**Figure:**
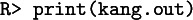

**Figure:**
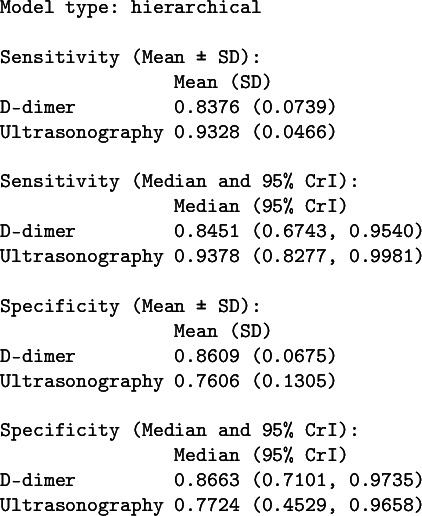
Long description.

As shown above, each list for the effect sizes in the object kang.out consists of posterior means with sample standard deviations specified in the parenthesis and posterior medians with 95% equal tail credible interval. These results indicate that, on average, Ultrasonography shows higher sensitivity (median = 0.94, 95% CrI: 0.83–1.00) but lower specificity (median = 0.77, 95% CrI: 0.45–0.97) compared with D-dimer. This suggests that Ultrasonography is more effective in identifying true positive cases but may yield more false positives than D-dimer, reflecting a trade-off between sensitivity and specificity that is typical in diagnostic accuracy analyses.

In addition to these test-specific summaries, NMADTA now provides direct relative comparisons between tests. Specifically, the output includes pairwise differences in sensitivity and specificity (returned as $dSe and $dSp), which are computed by subtracting posterior samples across MCMC iterations and summarized using the same posterior Mean(SD) and Median(95% CrI) format. For the current example with two tests, kang.out$dSe and kang.out$dSp quantify the magnitude and uncertainty of the sensitivity and specificity differences between Ultrasonography and D-dimer. A positive value of ΔSe (or ΔSp ) indicates higher sensitivity (or specificity) for the first-listed test, and a 95% CrI excluding zero provides strong posterior evidence of a meaningful difference under the fitted model.

**Figure:**
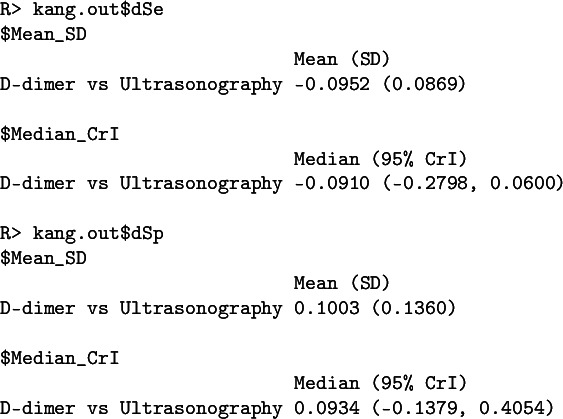
Long description.

In the example code, dic was specified as TRUE so the DIC statistic would be calculated. Users can access the DIC statistic and its components using

**Figure:**
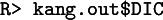

**Figure:**
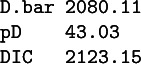

**Figure 1:**
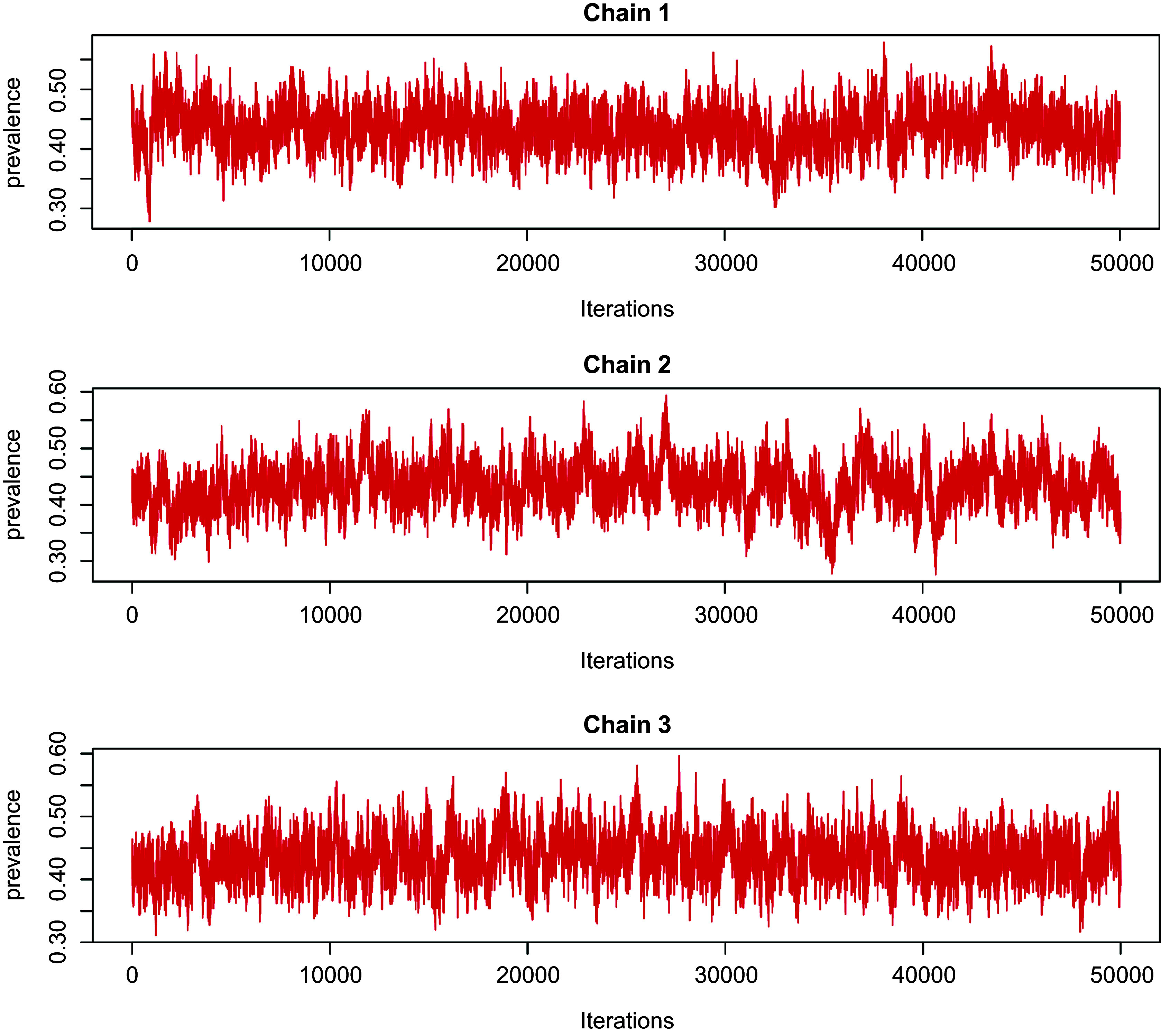
Trace plots generated by the R function nmadt.hierarchical for the prevalence of DVT in dat.kang Figure 1 long description.

Trace plots for prevalence were generated because trace = "prev" was specified. Figure 1 shows the trace plots of the prevalence of DVT in the case study. The argument trace is a string vector describing the subset of quantities for which trace plots can be drawn, which can include prevalence ("prev"), sensitivity ("Se"), specificity ("Sp"), positive and negative predictive values ("ppv" and "npv", respectively), positive likelihood ("LRpos"), and negative likelihood ("LRneg"). Since we used the default n.chains = 3, three trace plots are drawn. Each trace plot shows evidence that the posterior samples of prevalence are drawn from the stationary distribution. In addition to visual inspection of trace plots, NMADTA provides a quantitative convergence diagnostic based on the PSRF proposed by Gelman and Rubin. When conv.diag = TRUE, PSRF values are computed for the monitored parameters and saved in a text file (e.g., ConvergenceDiagnostic.txt) in the user-specified directory. The PSRF compares between-chain and within-chain variability, and values approaching 1 indicate convergence. In practice, PSRF values below 1.1 are commonly regarded as evidence of acceptable convergence, while values closer to 1.0 (e.g., < 1.05) provide stronger reassurance of adequate mixing.^38^ ^,^ ^41^ Upper confidence limits substantially above these thresholds may indicate lack of convergence and the need for additional iterations.

#### 4.2.2 Function nmadt.hierarchical.MNAR() under MNAR assumptions

In this context, MNAR refers specifically to outcome-dependent test performance availability within the evidence network. That is, the probability that a diagnostic test appears in a study (or that its accuracy results are reported) may depend on its underlying performance. This differs from standard MAR assumptions, under which test availability is assumed independent of unobserved accuracy parameters. While publication bias can be viewed as one manifestation of MNAR, our formulation more generally captures selection processes that operate at the test level within multi-test diagnostic studies. In the regular NMA-DT model, we assume that tests are missing from studies at random, but this assumption can be questionable in some situations. Sometimes tests can be MNAR, and we would need a different missingness mechanism for the meta-analysis. The function nmadt.hierarchical.MNAR permits a sensitivity analysis in which tests are MNAR. We define an N×K matrix M denoting the missingness status of the *K* candidate tests in each of the *N* studies. Entry $m_{ik}$ ( i=1,…,N , k=1,…,K ) of M indicates whether $T_{k}$ is missing in study *i*, that is, $m_{ik}=1$ if missing and $m_{ik}=0$ otherwise. We model $m_{ik}∼Bern(p_{ik})$ , where $p_{ik}$ is the probability that $T_{k}$ is missing in study *i*, and specify

(4.1) $$\begin{array}{r} \begin{array}{c} \mathrm{logit}(p_{ik})=\gamma_{k}+\gamma_{1k}\times \mathrm{logit}({\text{Se}}_{ik})+\gamma_{0k}\times \mathrm{logit}({\text{Sp}}_{ik}), \end{array} \end{array}$$

where $\gamma_{1k}$ and $\gamma_{0k}$ represent the strength of association between missingness and the study-specific sensitivity and specificity, respectively. Note that $\gamma_{1k}$ and $\gamma_{0k}$ are regression coefficients in the logistic missingness model on the latent scale, rather than correlation coefficients; therefore, they are not constrained to lie within [−1,1] .

The arguments of nmadt.hierarchical.MNAR are similar to those of nmadt.hierarchical. The major difference is that in nmadt.hierarchical.MNAR, users need to specify values of $\gamma_{1k}$ and $\gamma_{0k}$ , described above, as vectors of length *K* in the arguments gamma1 and gamma0. Users can also specify the prior mean and precision for $\gamma_{k}$ using mu_gamma and preci_gamma; the default is a vague N(0,10) . The dataset dat.kang is used again as an example:

**Figure:**
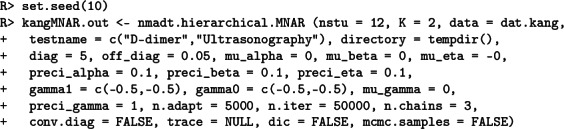
Long description.

For the dat.kang example, we set $\gamma_{1k}$ and $\gamma_{0k}$ to non-positive values to represent the assumption that $T_{k}$ is more likely to be missing if $T_{k}$ has low accuracy. In the MNAR missingness model, $\text{logit}(p_{ik})=\gamma_{k}+\gamma_{1k}\text{logit}({\mathrm{Se}}_{ik})+\gamma_{0k}\text{logit}({\mathrm{Sp}}_{ik})$ , $\gamma_{1k}$ and $\gamma_{0k}$ are logistic regression coefficients (not correlation parameters) and are therefore not constrained to [−1,1] . A useful interpretation is that $\exp(\gamma_{1k})$ (resp. $\exp(\gamma_{0k})$ ) is the multiplicative change in the odds of missingness associated with a one-unit increase in $\text{logit}({\mathrm{Se}}_{ik})$ (resp. $\text{logit}({\mathrm{Sp}}_{ik})$ ), holding other terms fixed. We recommend treating $\left(\gamma_{1k},\gamma_{0k}\right)$ as sensitivity-analysis parameters: (0,0) corresponds to MAR with respect to accuracy, while negative values (e.g., {0,−0.5,−1,−2} ) represent the common assumption that tests with poorer accuracy are more likely to be missing. Sensitivity analyses should be reported alongside routine convergence diagnostics. Here, more negative values imply a stronger MNAR dependence, that is, a larger increase in the odds of missingness as study-specific accuracy decreases on the logit scale. In practice, we recommend exploring a small grid, reporting how pooled sensitivity/specificity and relative test rankings change, and avoiding excessively large magnitudes that may push missingness probabilities toward 0/1 and slow MCMC mixing.

#### 4.2.3 Functions nmadt.hsroc() and nmadt.hsroc.MNAR() for the HSROC model

Functions nmadt.hsroc and nmadt.hsroc.MNAR implement the HSROC models introduced previously, respectively, under the MAR assumption and the MNAR assumption, under which a test’s missingness is believed to be related to its accuracy. The arguments of these two functions are similar to those of nmadt.hierarchical. The arguments eta and xi_precision specify the prior mean and precision of log⁡(S) and log⁡(P) , the covariance matrices of the cutoff values and accuracy values, respectively.

MNAR mechanisms for the HSROC analysis use a missingness model similar to the one used for the hierarchical analysis. For nmadt.hsroc.MNAR, as for nmadt.hierarchical.MNAR, users need to specify values of $\gamma_{1k}$ and $\gamma_{0k}$ , described above, as vectors of length *K* in arguments gamma1 and gamma0; they can also specify a prior mean and precision for $\gamma_{k}$ other than the default vague N(0,10) . Since the two functions are similar, and they give output in the same format as the analogous functions for the hierarchical models, we use the dataset dat.kang to illustrate the usage of nmadt.hsroc. The function is called as follows:

**Figure:**
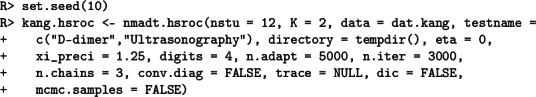
Long description.

Because the HSROC model is more complicated and has more parameters to estimate, the warning “adaptation incomplete” may occur more frequently. To avoid this warning, users can increase n.adapt.

It should be noted that the network diagnostic meta-analysis model is computationally intensive by design. This is primarily due to three factors. First, the model adopts a Bayesian hierarchical network structure and relies on MCMC sampling for inference, which requires joint sampling of high-dimensional random effects. Second, the incorporation of MNAR mechanisms introduces additional latent variables and regression parameters associated with the missingness process, substantially increasing model complexity. Third, jointly modeling multiple diagnostic tests across studies induces strong posterior dependence among parameters, which can slow down MCMC mixing and necessitate a larger number of iterations to achieve stable posterior inference. Consequently, longer runtime is generally expected for this model compared with standard diagnostic test meta-analysis models. For practical use, we recommend that users first run the model with a reduced number of MCMC iterations to verify data formatting and model behavior, and then increase the number of iterations for final analyses after convergence has been assessed. For illustration purposes and to reduce computation time, we use a smaller number of MCMC iterations (e.g., n.iter = 3000) in the example code. This setting is intended only to demonstrate the workflow and basic functionality. For real-world analyses, users should increase n.iter (and assess convergence diagnostics) to obtain stable posterior summaries. Corresponding analyses based on the HSROC model are provided in the Supplementary Material.

### 4.3 Plotting NMA-DT results

When presenting NMA results, it is helpful to show density plots of the posteriors of sensitivity and specificity as well as forest plots, SROC curves, and contour plots of SROC curves. The NMADTA package provides an S3 plotting method for fitted model objects, allowing users to generate a variety of diagnostic accuracy plots through a unified plot() interface. Specifically, setting the argument type to "sroc", "density", "forest", or "contour". Users call these functions for objects obtained from nmadt.hierarchical, nmadt.hierarchical.MNAR, nmadt.hsroc, and nmadt.hsroc.MNAR. Here, we use the kang.out object obtained from running nmadt.hierarchical in the previous section as an example:

**Figure:**
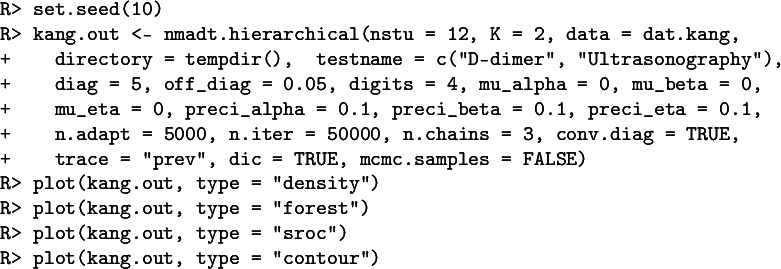
Long description.

The argument type is a string vector representing different types of plots which can be density plots ("density"), forest plots ("forest"), SROC curves ("sroc"), and contour plots of SROC curves ("contour").

**Figure 2:**
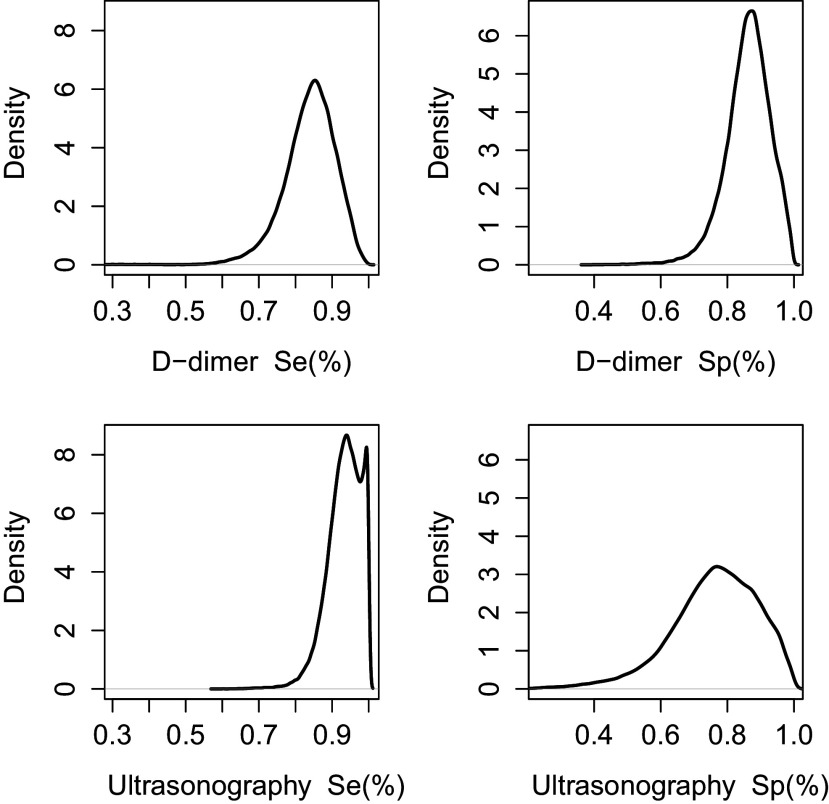
Density plots generated by the R function plot() applied to an nmadt object (with type = "density") for posterior true positive rates versus false positive rates for the diagnostic tests in dat.kang Figure 2 long description.

**Figure 3:**
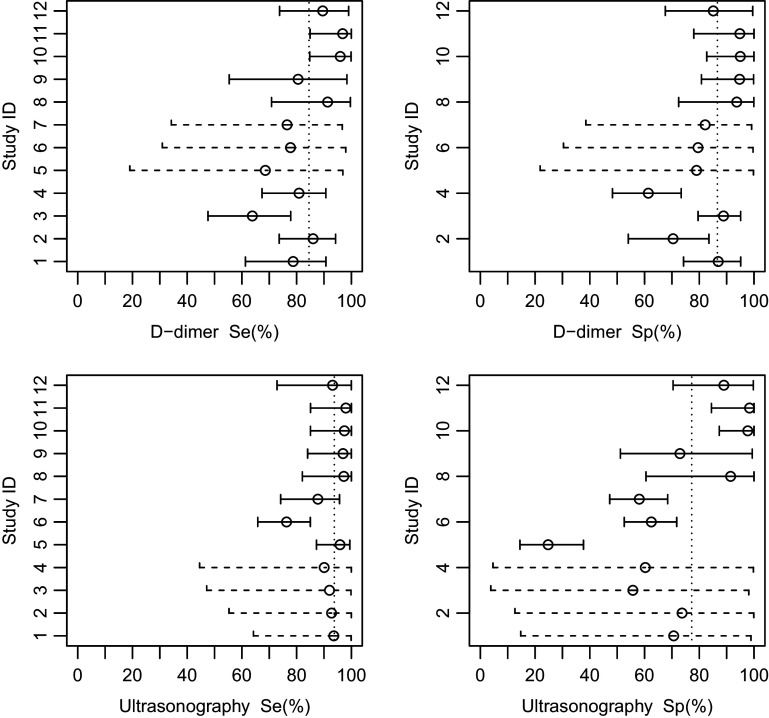
Forest plots generated by the R function plot() applied to an nmadt object (with type = "forest") for study-specific posterior sensitivities and specificities of the diagnostic tests in dat.kang Figure 3 long description.

**Figure 4:**
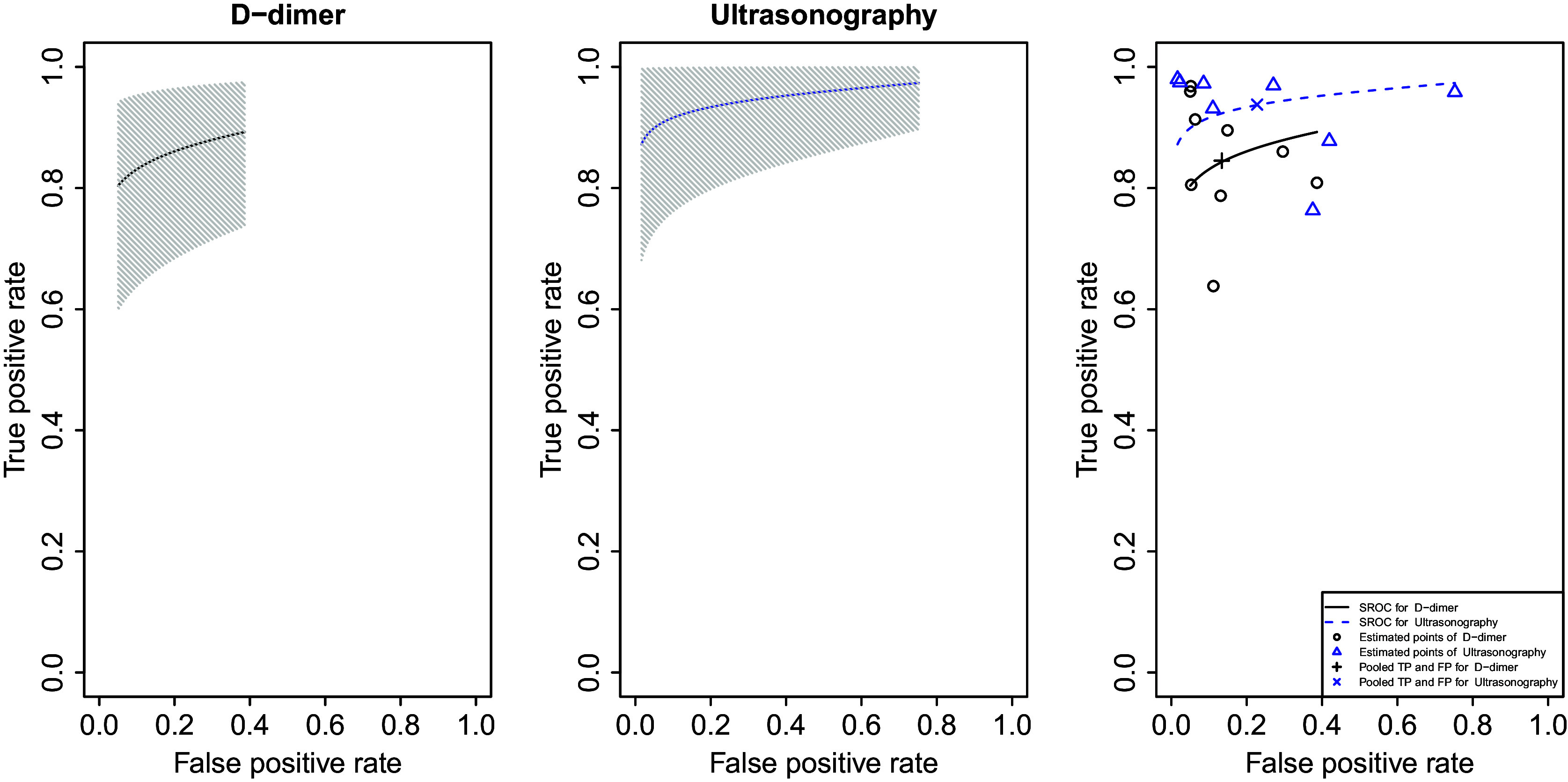
SROC curves and combined SROC plot generated by the R function plot() applied to an nmadt object (with type = "sroc") for posterior true positive rates versus false positive rates for the diagnostic tests in dat.kang, the left and middle panels display the posterior SROC curve for D-dimer and Ultrasonography, respectively (shaded areas indicate 95% credible bands). The right panel overlays the test-specific SROC curves and shows study-level operating points (sensitivity vs. 1–specificity) to illustrate between-study variability and the distribution of evidence across the ROC space Figure 4 long description.

**Figure 5:**
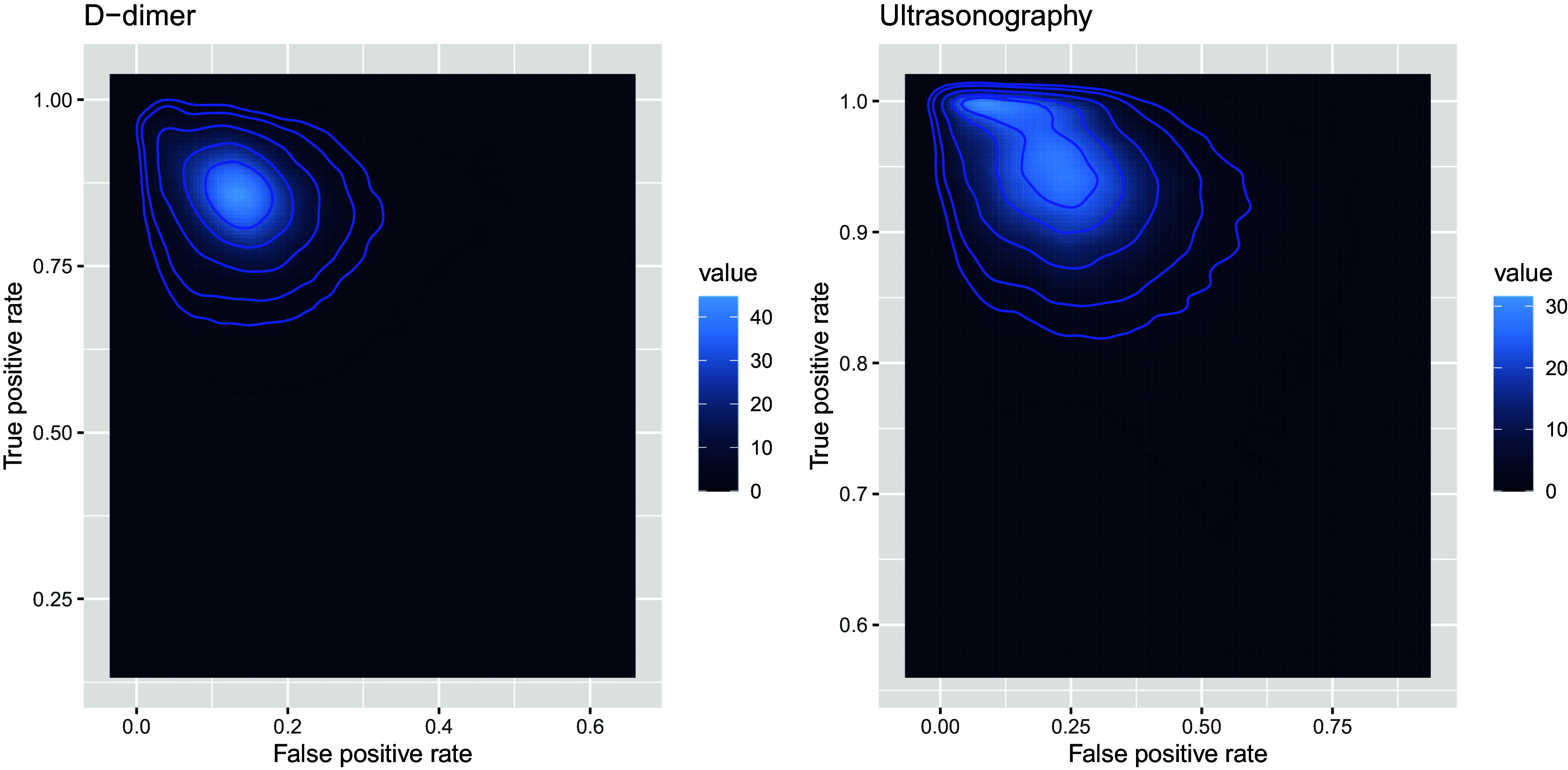
Contour plots generated by the R function plot() applied to an nmadt object (with type = "contour") for posterior true positive rates versus false positive rates for the diagnostic tests in dat.kang Figure 5 long description.

Figures 2–5 illustrate a step-by-step visualization of the hierarchical model results using NMADTA. Figure 2 presents the posterior density plots of test-specific sensitivity and specificity, showing that both parameters are well identified and approximately symmetric, suggesting stable MCMC convergence. Figure 3 displays the forest plots of study-level posterior medians and 95% credible intervals for the two diagnostic tests, where solid lines indicate directly observed studies and dashed lines indicate imputed results for missing tests. The results demonstrate moderate between-study variability, with ultrasonography generally showing higher sensitivity but lower specificity compared with D-dimer.

Figure 4 shows the hierarchical SROC curves, along with the observed and pooled true-positive and false-positive rates for each test. Ultrasonography achieves a steeper curve toward the upper left corner, indicating better discrimination performance on average. Finally, Figure 5 depicts the posterior quantile contour plots of the true-positive rate versus the false-positive rate at quantile levels 0.25, 0.5, 0.75, 0.90, and 0.95, summarizing the joint uncertainty structure in diagnostic accuracy. Together, these plots demonstrate how NMADTA enables comprehensive visualization and interpretation of NMA results under hierarchical models.

## 5 Summary and discussion

This article presents an overview of the R package NMADTA. The package implements both Bayesian hierarchical and Bayesian HSROC models for conducting NMA of up to five diagnostic tests simultaneously, with or without a gold standard test. The package also includes visualization tools to facilitate the presentation and interpretation of results, and its use is illustrated through real-world examples of NMA of diagnostic tests.

We highlight three issues that may affect practical use. First, identifiability can be challenging in Bayesian NMA models for diagnostic tests, particularly when the network is sparse (e.g., many studies evaluate only a subset of tests) or when the reference gold standard is frequently missing. In such settings, some variance–covariance components and correlation structures may be only weakly identified by the data, which can increase dependence on prior choices and adversely affect MCMC mixing. Routine convergence diagnostics and prior sensitivity analyses are therefore recommended. Although a logit link could be used in place of the probit link, we retain the probit formulation because it admits a convenient latent normal representation that tends to improve numerical stability and MCMC mixing in our hierarchical multivariate random-effects specification; in practice, probit and logit links typically yield very similar inferences (up to a scale difference on the transformed scale). Second, our MNAR implementation adopts a logistic selection model that links test missingness to latent, study-specific accuracy measures (e.g., ${\mathrm{Se}}_{ik}$ and ${\mathrm{Sp}}_{ik}$ ) to provide an interpretable and flexible sensitivity-analysis framework. This choice is not unique; alternative MNAR formulations (e.g., dependence on other latent components, study characteristics, or different link functions) may be considered when motivated by subject-matter knowledge, and we encourage sensitivity analyses across plausible specifications. Third, the missing-data mechanism is generally not identifiable from the observed data alone under MNAR assumptions without additional assumptions or external information (e.g., validation subsamples, repeated measurements, or design-based constraints). Consequently, MNAR analyses should be interpreted as structured sensitivity analyses that evaluate the robustness of key posterior summaries across a range of plausible missingness mechanisms.

An important direction for future research is the development of rigorous methods to evaluate and address publication bias in NMA of diagnostic test accuracy. Promising extensions include incorporating model-based selection mechanisms or weight-function approaches within hierarchical NMADTA frameworks, adapting regression-based small-study effect diagnostics to multivariate diagnostic accuracy outcomes, and developing sensitivity analysis tools that quantify the impact of alternative selective-reporting assumptions on network estimates. With continued methodological advances, such tools could be seamlessly integrated into the proposed software architecture, further enhancing the practical utility of the NMADTA package for applied researchers.

The current version of NMADTA does not handle meta-analyses comparing more than 5 diagnostic tests simultaneously. Future work could extend the current functions and add functions to conduct more complex NMA for diagnostic tests and to compute other visualization tools for assessing the performance of the MCMC. One potential approach might be using Bayesian composite likelihood to reduce the number of parameters to be estimated in the models, in order to handle the mentioned limitation of fewer than 5 tests. Also, the package currently only allows users to vary the values of the parameters but not the distributional form of the prior distributions; more work can be done to accommodate different prior forms. Currently, NMADTA only provides trace plots and PSRFs for convergence diagnostics, but these measures can sometimes be misleading.^42^ Finally, both models implemented in NMADTA are parametric; future work can extend these NMADT models to semi-parametric or non-parametric settings to accommodate more complicated study designs and scenarios in which normality cannot be assumed.

## Supporting information

10.1017/rsm.2026.10097.sm001 Xing et al. supplementary material Xing et al. supplementary material

## Data Availability

Users can download the source file of NMADTA at https://CRAN.R-project.org/package=NMADTA. Replication data and code are publicly available at https://osf.io/46jvc/.

## Supplementary material

The supplementary material for this article can be found at https://doi.org/10.1017/rsm.2026.10097.

## Long descriptions

### Long description

The code block consists of five lines.

Line 1: nmadt dot hierarchical open parenthesis n s t u, K, data, testname, directory equals N U L L,

Line 2: plus diag equals 5, off underscore diag equals 0.05, digits equals 4, mu underscore alpha equals 0, mu underscore beta equals 0,

Line 3: plus mu underscore eta equals minus 0, preci underscore alpha equals 0.1, preci underscore beta equals 0.1, preci underscore eta equals 0.1,

Line 4: plus n dot adapt equals 5000, n dot iter equals 50000, n dot chains equals 3,

Line 5: plus conv dot diag equals F A L S E, trace equals N U L L, dic equals F A L S E, m c m c dot samples equals F A L S E close parenthesis.

Navigate back to .

### Long description

The code consists of two main R commands.

Line 1: R prompt followed by set dot seed open parenthesis 10 close parenthesis.

Line 2 through 7: A multi-line function call assigning a value to the object kang dot out. The function is n m a d t dot hierarchical with the following arguments:

* n s t u equals 12

* K equals 2

* data equals dat dot kang

* directory equals tempdir open parenthesis close parenthesis

* testname equals c open parenthesis double quote D dash dimer double quote comma double quote Ultrasonography double quote close parenthesis

* diag equals 5

* off underscore diag equals 0.05

* digits equals 4

* mu underscore alpha equals 0

* mu underscore beta equals 0

* mu underscore eta equals 0

* preci underscore alpha equals 0.1

* preci underscore beta equals 0.1

* preci underscore eta equals 0.1

* n dot adapt equals 5000

* n dot iter equals 50000

* n dot chains equals 3

* conv dot diag equals TRUE

* trace equals double quote prev double quote

* dic equals TRUE

* m c m c dot samples equals FALSE

Navigate back to .

### Long description

The log begins with the line Start running M C M C dot dot dot followed by a series of indented processes.

Compiling data graph includes

* Resolving undeclared variables

* Allocating nodes

* Initializing

* Reading data back into data table

Compiling model graph includes

* Resolving undeclared variables

* Allocating nodes

Graph information provides the following data

* Observed stochastic nodes 48

* Unobserved stochastic nodes 18

* Total graph size 1898

Initializing model is followed by three progress bars. The first is filled with plus signs and marked 100 percent. The second and third are filled with asterisks and marked 100 percent.

The final section lists three concluding actions

* Start calculating M C M C convergence diagnostic statistics dot dot dot

* Start calculating deviance information criterion statistics dot dot dot followed by a progress bar of asterisks at 100 percent

* Start saving trace plots dot dot dot

Navigate back to .

### Long description

The document begins with the header Model type hierarchical. It is divided into four sections.

1. Sensitivity Mean plus or minus S D.

* D-dimer has a mean of 0.8376 and an S D of 0.0739.

* Ultrasonography has a mean of 0.9328 and an S D of 0.0466.

2. Sensitivity Median and 95 percent C r I.

* D-dimer has a median of 0.8451 with a 95 percent C r I of 0.6743 to 0.9540.

* Ultrasonography has a median of 0.9378 with a 95 percent C r I of 0.8277 to 0.9981.

3. Specificity Mean plus or minus S D.

* D-dimer has a mean of 0.8609 and an S D of 0.0675.

* Ultrasonography has a mean of 0.7606 and an S D of 0.1305.

4. Specificity Median and 95 percent C r I.

* D-dimer has a median of 0.8663 with a 95 percent C r I of 0.7101 to 0.9735.

* Ultrasonography has a median of 0.7724 with a 95 percent C r I of 0.4529 to 0.9658.

Navigate back to .

### Long description

The output is divided into two main sections based on R commands.

First section: R greater than kang dot out dollar d S e.

This section contains two sub-blocks for sensitivity data.

1. Under the header dollar Mean underscore S D, the row D-dimer vs Ultrasonography shows a Mean of negative 0.0952 and a S D of 0.0869.

2. Under the header dollar Median underscore C r I, the row D-dimer vs Ultrasonography shows a Median of negative 0.0910 and a 95 percent C r I ranging from negative 0.2798 to 0.0600.

Second section: R greater than kang dot out dollar d S p.

This section contains two sub-blocks for specificity data.

1. Under the header dollar Mean underscore S D, the row D-dimer vs Ultrasonography shows a Mean of 0.1003 and a S D of 0.1360.

2. Under the header dollar Median underscore C r I, the row D-dimer vs Ultrasonography shows a Median of 0.0934 and a 95 percent C r I ranging from negative 0.1379 to 0.4054.

Navigate back to .

### Figure 1 Long description

A multi-panel figure containing three vertically stacked line graphs labeled Chain 1, Chain 2, and Chain 3. All three graphs share identical axes. The horizontal x-axis represents Iterations, ranging from 0 to 50,000 with major ticks every 10,000. The vertical y-axis represents prevalence, ranging from 0.30 to 0.60.

* Chain 1 at the top shows a red jagged line fluctuating rapidly between 0.35 and 0.55. The trace appears stable with no significant upward or downward trend, indicating convergence.

* Chain 2 in the middle displays a similar red trace plot. The density of the fluctuations is consistent across the 50,000 iterations, centered around a prevalence of approximately 0.42.

* Chain 3 at the bottom mirrors the behavior of the previous two chains, with the red line oscillating within the same range of 0.30 to 0.60.

All three chains exhibit a fuzzy caterpillar appearance, which is characteristic of well-mixed Markov Chain Monte Carlo simulations.

Navigate back to Figure 1.

### Long description

The code begins with the command set dot seed 10. The main function call assigns the result to kang M N A R dot out using the function nmadt dot hierarchical dot M N A R. The function parameters are listed across several lines starting with a plus sign.

Line 1 includes nstu equals 12, K equals 2, and data equals dat dot kang.

Line 2 defines testname as a vector containing D-dimer and Ultrasonography, and directory as tempdir.

Line 3 sets diag equals 5, off underscore diag equals 0.05, mu underscore alpha equals 0, mu underscore beta equals 0, and mu underscore eta equals negative 0.

Line 4 sets preci underscore alpha, preci underscore beta, and preci underscore eta all to 0.1.

Line 5 sets gamma 1 and gamma 0 to vectors of negative 0.5 and negative 0.5, and mu underscore gamma equals 0.

Line 6 sets preci underscore gamma equals 1, n dot adapt equals 5000, n dot iter equals 50000, and n dot chains equals 3.

Line 7 concludes with conv dot diag equals FALSE, trace equals NULL, dic equals FALSE, and mcmc dot samples equals FALSE.

Navigate back to .

### Long description

The code consists of two main commands.

First line: R prompt followed by set dot seed open parenthesis 10 close parenthesis.

Second line through sixth line: A multi-line function call assigned to the variable kang dot h s r o c.

- The function is n m a d t dot h s r o c with the following arguments:

- n s t u equals 12

- K equals 2

- data equals dat dot kang

- testname equals c open parenthesis double quote D dash dimer double quote comma double quote Ultrasonography double quote close parenthesis

- directory equals tempdir open parenthesis close parenthesis

- eta equals 0

- xi underscore preci equals 1.25

- digits equals 4

- n dot adapt equals 5000

- n dot iter equals 3000

- n dot chains equals 3

- conv dot diag equals FALSE

- trace equals NULL

- dic equals FALSE

- m c m c dot samples equals FALSE

Navigate back to .

### Figure 2 Long description

A multi-panel figure containing four density plots arranged in a two by two grid. All plots share a vertical Y axis labeled Density.

* Top-left panel: The X axis is labeled D-dimer S e percent ranging from 0.3 to 1.0. The curve shows a unimodal distribution with a peak density of approximately 6 at an S e value of 0.85.

* Top-right panel: The X axis is labeled D-dimer S p percent ranging from 0.4 to 1.0. The curve shows a narrow unimodal distribution peaking at a density of approximately 6.5 at an S p value of 0.88.

* Bottom-left panel: The X axis is labeled Ultrasonography S e percent ranging from 0.3 to 1.0. The curve shows a bimodal distribution with a primary peak at 0.95 and a secondary peak near 1.0, reaching a maximum density of approximately 8.5.

* Bottom-right panel: The X axis is labeled Ultrasonography S p percent ranging from 0.4 to 1.0. The curve shows a broad, lower-density unimodal distribution peaking at approximately 3.2 at an S p value of 0.78.

Navigate back to Figure 2.

### Figure 3 Long description

A multi-panel figure containing four forest plots arranged in a two-by-two grid. Each plot features a vertical y-axis labeled Study I D with values from 1 to 12 and an x-axis representing percentage from 0 to 100. A vertical dotted line indicates the overall mean estimate.

Top-Left Panel: D-dimer S e percent. Data points for studies 1 through 12 show sensitivity values generally clustered between 60 and 100 percent. Studies 5, 6, and 7 are represented by dashed horizontal error bars, while others use solid lines. Study 9 has the widest error bar extending down to 55 percent.

Top-Right Panel: D-dimer S p percent. Specificity values for D-dimer. Studies 5, 6, and 7 again use dashed lines. Most studies cluster between 70 and 100 percent, though Study 4 shows a lower mean near 60 percent.

Bottom-Left Panel: Ultrasonography S e percent. Sensitivity values for ultrasonography. Studies 1 through 4 use dashed error bars and show high sensitivity near 95 percent. Studies 5 through 12 use solid lines, with Study 6 showing the lowest mean sensitivity in this group at approximately 75 percent.

Bottom-Right Panel: Ultrasonography S p percent. Specificity values for ultrasonography. Studies 1 through 4 use dashed lines and show a wide range of specificity. Studies 5 through 12 use solid lines, with Study 5 showing the lowest specificity near 25 percent and Study 10 showing the highest near 98 percent.

Navigate back to Figure 3.

### Figure 4 Long description

The multi-panel line graph consists of three side-by-side plots with identical axes. The x-axis represents the False positive rate from 0.0 to 1.0, and the y-axis represents the True positive rate from 0.0 to 1.0.

* The left panel, titled D-dimer, shows a black dotted S R O C curve starting at approximately 0.05 on the x-axis and 0.8 on the y-axis, rising with a slight concave shape toward 0.4 on the x-axis and 0.9 on the y-axis. A grey hatched area represents the 95 percent credible band surrounding the curve.

* The middle panel, titled Ultrasonography, shows a blue dotted S R O C curve starting near 0.0 on the x-axis and 0.85 on the y-axis, rising more steeply and plateauing toward 0.8 on the x-axis and 0.98 on the y-axis. A grey hatched area represents its 95 percent credible band.

* The right panel overlays both tests and includes study-level data. A solid black line represents the S R O C for D-dimer, accompanied by open circles for estimated points and a plus sign for the pooled T P and F P. A dashed blue line represents the S R O C for Ultrasonography, accompanied by open triangles for estimated points and an x mark for the pooled T P and F P. The Ultrasonography curve sits higher in the top-left corner than the D-dimer curve, indicating higher diagnostic accuracy. A legend in the bottom-right corner identifies all six symbols and line types.

Navigate back to Figure 4.

### Figure 5 Long description

A two-panel attribute space diagram.

Left Panel: Titled D-dimer. The x-axis represents False positive rate from 0.0 to 0.6. The y-axis represents True positive rate from 0.25 to 1.00. A color scale legend to the right indicates values from 0 to 40. At the core, a bright light-blue peak is centered at approximately 0.15 on the x-axis and 0.85 on the y-axis. Moving outward, concentric blue contour lines expand to cover a range between 0.0 and 0.4 on the x-axis and 0.65 to 1.0 on the y-axis.

Right Panel: Titled Ultrasonography. The x-axis represents False positive rate from 0.00 to 0.75. The y-axis represents True positive rate from 0.6 to 1.0. A color scale legend to the right indicates values from 0 to 30. At the core, a bright light-blue peak is located near 0.15 on the x-axis and 0.95 on the y-axis. The next layers outward show contour lines stretching horizontally toward 0.5 on the x-axis and vertically down to 0.8 on the y-axis, indicating a wider distribution of posterior rates compared to the first panel.

Navigate back to Figure 5.

### Long description

The code begins with setting a seed for reproducibility using set dot seed 10.

A model object named kang dot out is created using the function n m a d t dot hierarchical with the following arguments.

* n s t u equals 12.

* K equals 2.

* data equals dat dot kang.

* directory equals tempdir.

* testname equals a vector containing D dash dimer and Ultrasonography.

* diag equals 5.

* off underscore diag equals 0.05.

* digits equals 4.

* mu underscore alpha, mu underscore beta, and mu underscore eta all equal 0.

* preci underscore alpha, preci underscore beta, and preci underscore eta all equal 0.1.

* n dot adapt equals 5000.

* n dot iter equals 50000.

* n dot chains equals 3.

* conv dot diag equals T R U E.

* trace equals prev.

* d i c equals T R U E.

* m c m c dot samples equals F A L S E.

The script concludes with four plot commands for the kang dot out object with types specified as density, forest, s r o c, and contour.

Navigate back to .
