# Supplementary material for: NMADTA: An R package for network meta-analysis of multiple diagnostic tests
Source: Res Synth Methods. 2026 Jun 15:1–22. Online ahead of print. doi: 10.1017/rsm.2026.10097 (PMC13312819; doi:10.1017/rsm.2026.10097)
Supplement: Xing et al. supplementary material [file S1759287926100970sup001.pdf]

Supplementary Material for "NMADTA: An R Package for Network Meta-Analysis of Multiple Diagnostic Tests"

1 Examples of Using nmadt.hrsoc

The function `nmadt.hrsoc` returns a list with the raw output for graphing the results, the effect size estimates, including the posterior mean, standard deviation, median, and a 95% equal-tailed credible interval for the median.

```
R> set.seed(10)
R> kang.hsroc <- nmadt.hsroc(nstu = 12, K = 2, data = dat.kang,
+ testname = c("D-dimer","Ultrasonography"), directory = tempdir(),
+ eta = 0,  xi_preci = 1.25, digits = 4, n.adapt = 5000,
+ n.iter = 50000,  n.chains = 3, conv.diag = FALSE, trace = TRUE,
+ dic = FALSE, mcmc.samples = FALSE)
```

The following messages are output as the function runs:

```
Start running MCMC...
Compiling data graph
   Resolving undeclared variables
   Allocating nodes
   Initializing
   Reading data back into data table
Compiling model graph
   Resolving undeclared variables
   Allocating nodes
Graph information:
   Observed stochastic nodes: 48
   Unobserved stochastic nodes: 38
   Total graph size: 2232

Initializing model

  |++++++++++++++++++++++++++++++++++++++++++++++++++| 100%
  |++++++++++++++++++++++++++++++++++++++++++++++++++| 100%
NOTE: Stopping adaptation

  |**************************************************| 100%
Start calculating MCMC convergence diagnostic statistics...
Start calculating deviance information criterion statistics...
  |**************************************************| 100%
Start saving trace plots...
Warning message:
In jags.model(model, data = data.jags, inits = init, n.chains = n.chains,
Adaptation incomplete
```

When the proposed model is compiled, it requires an initial sampling phase during which the samplers adapt their behavior to maximize their efficiency. If the warning "adaptation incomplete" occurs, the users might need to increase `n.adapt` to make the process reach its maximum efficiency.

The results are saved in the list object `kang.hsroc`. It contains the raw output for prevalence, sensitivity, specificity, positive and negative predictive values, positive likelihood, negative likelihood, and DIC. The trace plots are saved in the user-specified directory. The effect size names can be used to display the estimates. For example, the estimates of sensitivities and specificities (posterior mean and standard deviation, and posterior median and 95% credible intervals) can be displayed as

```
R> print(kang.hsroc)

Model type: HSROC

Sensitivity (Mean ± SD):
                Mean (SD)
D-dimer         0.8430 (0.0826)
Ultrasonography 0.9476 (0.0620)

Sensitivity (Median and 95% CrI):
                Median (95% CrI)
D-dimer         0.8477 (0.6627, 0.9856)
Ultrasonography 0.9633 (0.8092, 1.0000)

Specificity (Mean ± SD):
                Mean (SD)
D-dimer         0.8714 (0.0782)
Ultrasonography 0.8278 (0.1584)

Specificity (Median and 95% CrI):
                Median (95% CrI)
D-dimer         0.8780 (0.6968, 0.9940)
Ultrasonography 0.8603 (0.4243, 1.0000)
```

As shown above, the summary for each diagnostic accuracy measure includes posterior means with sample standard deviations (in parentheses), as well as posterior medians with 95% equal-tail credible intervals (CrIs). Under the HSROC model, Ultrasonography demonstrates higher sensitivity than D-dimer (median = 0.9633, 95% CrI: 0.8092–1.0000 vs. median = 0.8477, 95% CrI: 0.6627–0.9856). In terms of specificity, the posterior medians are very similar between the two tests (Ultrasonography: median = 0.8603, 95% CrI: 0.4243–1.0000; D-dimer: median = 0.8780, 95% CrI: 0.6968–0.9940), although the credible interval for Ultrasonography is notably wider, indicating greater uncertainty in specificity. Overall, these results suggest that Ultrasonography may be more effective in identifying true positive cases, while its specificity is comparable on average but less precisely estimated, reflecting the typical sensitivity–specificity trade-off in diagnostic accuracy analyses.

Trace plots for prevalence were generated because `trace = "prev"` was specified. Figure 1 shows the trace plots of the prevalence of DVT in the case study. Since we used the default `n.chains = 3`, three trace plots are drawn. Each trace plot shows evidence that the posterior samples of prevalence are drawn from the stationary distribution.

```
R> kang.out$dSe
$Mean_SD
                            Mean (SD)
D-dimer vs Ultrasonography -0.1046 (0.1015)

$Median_CrI
                            Median (95% CrI)
D-dimer vs Ultrasonography -0.1057 (-0.2989, 0.0864)
```

```
R> kang.out$dSp
$Mean_SD
                           Mean (SD)
D-dimer vs Ultrasonography 0.0436 (0.1695)

$Median_CrI
                           Median (95% CrI)
D-dimer vs Ultrasonography 0.0169 (-0.2154, 0.4418)
```

In the example code, `dic` was specified as TRUE so the DIC statistic would be calculated. Users can access the DIC statistic and its components using:

```
R> kang.hsroc$DIC

D.bar 2087.74
pD    30.82
DIC   2118.56
```

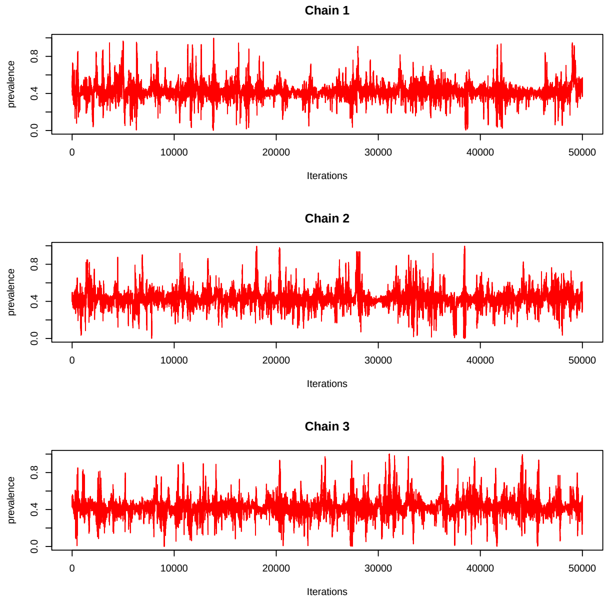

Figure 1: Trace plots generated by R function `nmadt.hsroc` for the prevalence of DVT in `dat.kang`.

2 Plotting NMA-DT results

When presenting network meta-analysis results, it is helpful to show density plots of the posteriors of sensitivity and specificity as well as forest plots, SROC curves, and contour plots of SROC curves. The `NMADTA` package provides an S3 plotting method for fitted model objects, allowing users to generate a variety of diagnostic accuracy plots through a unified `plot()` interface. Specifically, setting the argument `type` to `"sroc"`, `"density"`, `"forest"`, or `"contour"` . Users call these functions for objects obtained from `nmadt.hierarchical`, `nmadt.hierarchical.MNAR`, `nmadt.hsroc`, and `nmadt.hsroc.MNAR`. Here, we use the `kang.hsroc` object obtained from running `nmadt.hsroc` in the previous section as an example:

```
R> set.seed(10)
R> kang.hsroc <- nmadt.hsroc(nstu = 12, K = 2, data = dat.kang,
```

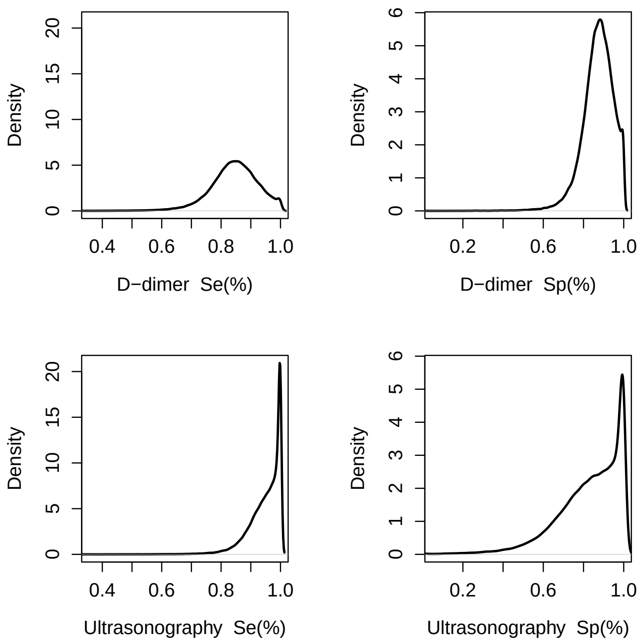

Figure 2: Density plots generated by the R function `plot()` applied to a `nmadt` object (with `type = "density"`) for posterior true positive rates versus false positive rates for the diagnostic tests in `dat.kang`.

```
+ testname = c("D-dimer","Ultrasonography"), directory = tempdir(),
+ eta = 0,  xi_preci = 1.25, digits = 4, n.adapt = 5000,
+ n.iter = 50000,  n.chains = 3, conv.diag = FALSE, trace = TRUE,
+ dic = FALSE, mcmc.samples = FALSE)
R> plot(kang.hsroc, type = "density")
R> plot(kang.hsroc, type = "forest")
R> plot(kang.hsroc, type = "sroc")
R> plot(kang.hsroc, type = "contour")
```

The argument `type` is a string vector representing different types of plots which can be density plots (`"density"`), forest plots (`"forest"`), SROC curves (`"sroc"`), and contour plots of SROC curves (`"contour"`).

Figures 2–5 illustrate a step-by-step visualization of the HSROC-based network meta-analysis results using `NMADTA`. Figure 2 presents the posterior density plots of test-specific sensitivity and specificity, showing that both parameters are well identified and approximately symmetric, suggesting stable MCMC convergence under the HSROC model. Figure 3 displays the forest plots of study-level posterior medians and 95% credible intervals for the two diagnostic tests, where solid lines indicate directly observed studies and dashed lines indicate imputed results for missing tests. The results demonstrate moderate between-study variability, with ultrasonography generally showing higher sensitivity while exhibiting similar or slightly lower specificity compared with D-dimer.

Figure 4 shows the HSROC curves together with observed and pooled operating points for each test, summarizing the average diagnostic performance across studies. Ultrasonography exhibits a steeper summary curve toward the upper-left corner of the ROC space, indicating better overall discrimination on average. Finally, Figure 5 depicts posterior quantile contour plots of the true-positive rate versus the false-positive rate at quantile levels 0.25, 0.5, 0.75, 0.90, and 0.95, summarizing the joint posterior uncertainty in diagnostic accuracy. Together, these plots demonstrate how `NMADTA` enables comprehensive visualization and interpretation of network meta-analysis results under the HSROC modeling framework.

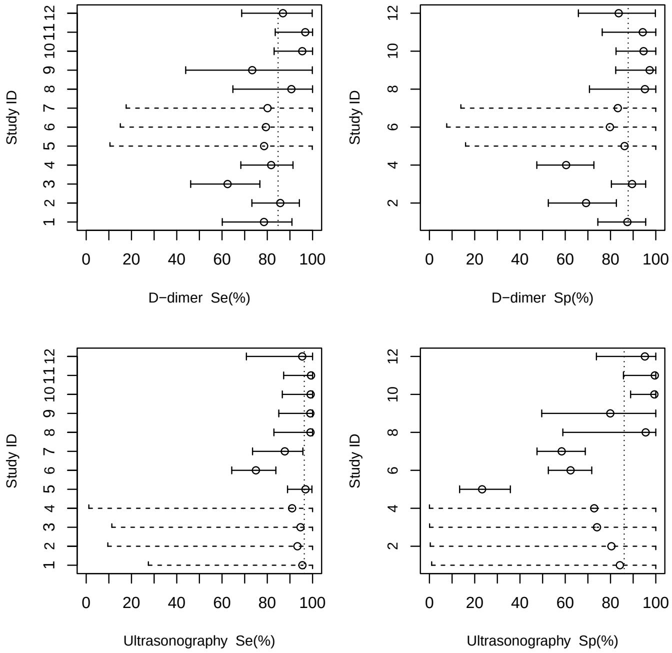

Figure 3: Forest plots generated by the R function `plot()` applied to a `nmadt` object (with `type = "forest"`) for study-specific posterior sensitivities and specificities of the diagnostic tests in `dat.kang`.

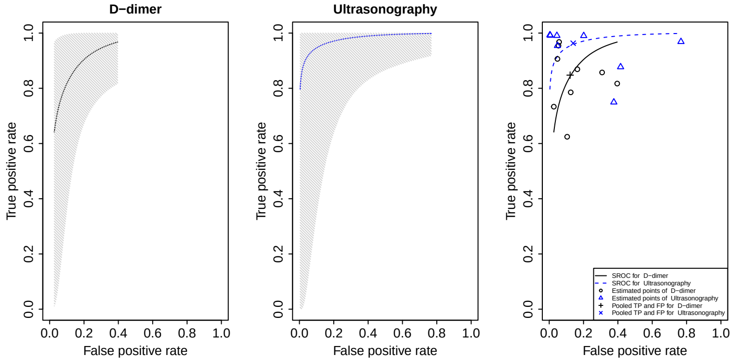

Figure 4: SROC curves and combined SROC plot generated by the R function `plot()` applied to a `nmadt` object (with `type = "sroc"`) for posterior true positive rates versus false positive rates for the diagnostic tests in `dat.kang`. The left and middle panels display the posterior SROC curve for D-dimer and Ultrasonography, respectively (shaded areas indicate 95% credible bands). The right panel overlays the test-specific SROC curves and shows study-level operating points (sensitivity vs. 1–specificity) to illustrate between-study variability and the distribution of evidence across the ROC space.

3 Function nmadt.hsroc.MNAR() under MNAR assumptions

In the regular HSROC network meta-analysis model, we typically assume that tests are missing from studies at random (MAR). However, this assumption may be questionable when the availability of a candidate test depends on its (latent) diagnostic performance in a study. To facilitate sensitivity analyses under missing-not-at-random (MNAR) mechanisms, the function `nmadt.hsroc.MNAR` extends the HSROC model by explicitly modeling the missingness indicator. Let $\mathbf{M}$ be an $N \times K$ matrix denoting the missingness status of the K candidate tests across N studies, where $m_{ik} = 1$ if test T_k is missing in study i and $m_{ik} = 0$ otherwise. We assume $m_{ik} \sim \text{Bern}(p_{ik})$ and link the missingness

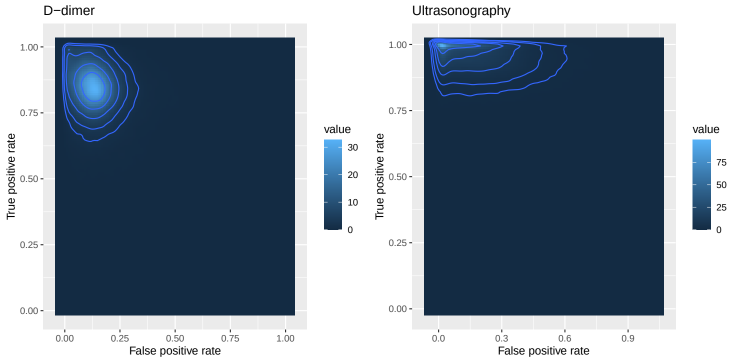

Figure 5: Contour plots generated by the R function `plot()` applied to a `nmadt` object (with `type = "contour"`) for posterior true positive rates versus false positive rates for the diagnostic tests in `dat.kang`.

probability to the study-specific sensitivity and specificity implied by the HSROC model via

$$\operatorname{logit}(p_{ik}) = \gamma_k + \gamma_{1k} \operatorname{logit}(\mathrm{Se}_{ik}) + \gamma_{0k} \operatorname{logit}(\mathrm{Sp}_{ik}), \tag{1}$$

The dataset `dat.kang` is used again as an example:

```
R> data(dat.kang)
R> set.seed()
R> kangMNAR.out.hsroc <- nmadt.hsroc.MNAR(nstu = 12, K = 2, data = dat.kang,
+   testname = c("D-dimer","Ultrasonography"),
+   gamma1 = c(-0.5, -0.5), gamma0 = c(-0.5, -0.5),
+   mu_gamma = 0, preci_gamma = 1,
+   n.adapt = 5000, n.iter = 50000, n.chains = 3,
+   conv.diag = FALSE, trace = NULL, dic = FALSE, mcmc.samples = FALSE)
```

where γ_{1k} and γ_{0k} quantify the dependence of missingness on (latent) sensitivity and specificity, respectively. The arguments of `nmadt.hsroc.MNAR` are similar to those of `nmadt.hsroc`; the key difference is that users must specify γ_{1k} and γ_{0k} as length-K vectors in `gamma1` and `gamma0` to encode plausible MNAR scenarios. Users may also specify the prior mean and precision for the intercept γ_k through `mu_gamma` and `preci_gamma`. In practice, we recommend treating $(\gamma_{1k}, \gamma_{0k})$ as sensitivity-analysis parameters: $(0, 0)$ corresponds to MAR with respect to accuracy, whereas negative values represent the common assumption that tests with poorer accuracy are more likely to be missing. Sensitivity analyses should be reported alongside routine convergence diagnostics, and a small grid of plausible values can be explored to assess the robustness of pooled accuracy estimates and relative test rankings.
